# Bias in machine learning applications to address non-communicable diseases at a population-level: a scoping review

**DOI:** 10.1186/s12889-024-21081-9

**Published:** 2024-12-28

**Authors:** Sharon Birdi, Roxana Rabet, Steve Durant, Atushi Patel, Tina Vosoughi, Mahek Shergill, Christy Costanian, Carolyn P. Ziegler, Shehzad Ali, David Buckeridge, Marzyeh Ghassemi, Jennifer Gibson, Ava John-Baptiste, Jillian Macklin, Melissa McCradden, Kwame McKenzie, Sharmistha Mishra, Parisa Naraei, Akwasi Owusu-Bempah, Laura Rosella, James Shaw, Ross Upshur, Andrew D. Pinto

**Affiliations:** 1 Upstream Lab, MAP Centre for Urban Health Solutions, Li Ka Shing Knowledge Institute, Unity Health Toronto, 30 Bond Street, Toronto, ON M5B 1W8 Canada; 2 Michael G. DeGroote School of Medicine, McMaster University, Hamilton, ON Canada; 3 Department of Family and Community Medicine, St. Michael’s Hospital, Toronto, ON Canada; 4 Department of Family and Community Medicine, Faculty of Medicine, University of Toronto, Toronto, ON Canada; 5 Division of Clinical Public Health, Dalla Lana School of Public Health, University of Toronto, Toronto, ON Canada; 6 Department of Epidemiology and Biostatistics, Western Centre for Public Health & Family Medicine, Western University, London, ON Canada; 7 Department of Epidemiology, Biostatistics and Occupational Health, School of Population and Global Health, McGill University, Montreal, QC Canada; 8 Department of Electrical Engineering and Computer Science (EECS) and Institute for Medical Engineering & Science (IMES), MIT, Cambridge, MA USA; 9 Joint Centre for Bioethics, University of Toronto, Toronto, ON Canada; 10 Departments of Epidemiology & Biostatistics, Anesthesia & Perioperative Medicine, Schulich Interfaculty Program in Public Health, Western University, London, ON Canada; 11 Undergraduate Medical Education, Faculty of Medicine, University of Toronto, Toronto, ON Canada; 12 Department of Bioethics, The Hospital for Sick Children, Toronto, ON Canada; 13 Genetics & Genome Biology, SickKids Research Institute, Toronto, ON Canada; 14 Wellesley Institute, Toronto, ON Canada; 15 CAMH, Toronto, ON Canada; 16 Division of Infectious Diseases, Department of Medicine, Faculty of Medicine, University of Toronto, Toronto, ON Canada; 17 MAP Centre for Urban Health Solutions, Li Ka Shing Knowledge Institute, Unity Health Toronto, Toronto, ON Canada; 18 Institute of Medical Science, Faculty of Medicine, University of Toronto, Toronto, Canada; 19 Institute of Health Policy, Management and Evaluation, Division of Epidemiology, Dalla Lana School of Public Health, University of Toronto, Toronto, Canada; 20 ICES, Toronto, ON Canada; 21 Department of Computer Science, Toronto Metropolitan University, Toronto, ON Canada; 22 Department of Sociology, Faculty of Arts & Sciences, University of Toronto, Toronto, ON Canada; 23 Institute for Better Health, Trillium Health Partners, Toronto, ON Canada; 24 Department of Physical Therapy, Faculty of Medicine, University of Toronto, Toronto, ON Canada; 25 Library Services, Unity Health Toronto, St. Michael’s Hospital, Toronto, ON Canada; 26 Division of Epidemiology, Dalla Lana School of Public Health, Toronto, ON Canada; 27 Department of Laboratory Medicine and Pathobiology, Temerty Faculty of Medicine, Toronto, ON Canada; 28 Department of Health Sciences, University of York, York, UK; 29 WHO Collaborating Centre for Knowledge Translation and Health Technology Assessment in Health Equity, Ottawa Centre for Health Equity, Ottawa, ON Canada

**Keywords:** Population health, Non-communicable disease, Machine learning, Artificial intelligence

## Abstract

**Background:**

Machine learning (ML) is increasingly used in population and public health to support epidemiological studies, surveillance, and evaluation. Our objective was to conduct a scoping review to identify studies that use ML in population health, with a focus on its use in non-communicable diseases (NCDs). We also examine potential algorithmic biases in model design, training, and implementation, as well as efforts to mitigate these biases.

**Methods:**

We searched the peer-reviewed, indexed literature using Medline, Embase, Cochrane Central Register of Controlled Trials and Cochrane Database of Systematic Reviews, CINAHL, Scopus, ACM Digital Library, Inspec, Web of Science’s Science Citation Index, Social Sciences Citation Index, and the Emerging Sources Citation Index, up to March 2022.

**Results:**

The search identified 27 310 studies and 65 were included. Study aims were separated into algorithm comparison (*n* = 13, 20%) or disease modelling for population-health-related outputs (*n* = 52, 80%). We extracted data on NCD type, data sources, technical approach, possible algorithmic bias, and jurisdiction. Type 2 diabetes was the most studied NCD. The most common use of ML was for risk modeling. Mitigating bias was not extensively addressed, with most methods focused on mitigating sex-related bias.

**Conclusion:**

This review examines current applications of ML in NCDs, highlighting potential biases and strategies for mitigation. Future research should focus on communicable diseases and the transferability of ML models in low and middle-income settings. Our findings can guide the development of guidelines for the equitable use of ML to improve population health outcomes.

**Supplementary Information:**

The online version contains supplementary material available at 10.1186/s12889-024-21081-9.

## Background

 Non-communicable diseases (NCDs), which include cardiovascular diseases, diabetes, cancers, and chronic respiratory diseases, are the leading cause of both burden of disease and death, globally, with a disproportionally higher rate of mortality in low- and middle-income countries (LMICs) [1, 2]. Population-level approaches of strengthening screening and detection are critical for identifying populations at high-risk of NCDs and informing early interventions [1]. One potential avenue for streamlining these interventions and lessening the burden of NCDs on the global population, is through artificial intelligence (AI) [3]. 

The increasing global interest in AI, particularly machine learning (ML), stems from the availability of large datasets and ever-growing computational power [4]. With its ability to learn and adapt from experience without explicit programming, ML has become crucial in various fields, such as healthcare [5]. However, alongside its remarkable potential, there are significant concerns associated with the widespread adoption of ML, notably the potential for algorithmic bias. *Algorithmic bias* in the context of AI and health systems is defined as: “the instances when the application of an algorithm compounds existing inequities in socioeconomic status, race, ethnic background, religion, gender, disability or sexual orientation to amplify them and adversely impact inequities in health systems” [6]. These biases, rooted in historical and systemic inequities, persistently affect marginalized groups, which reinforce prejudices. Marginalized groups, referring to individuals or communities who experience social, economic, or political disadvantages and discrimination, often bear the brunt of these amplified inequities in access to healthcare services and outcomes [7]. Reinforcing prejudices in this context means that predictive models, when trained on biased data or making decisions that align with historical disparities, inadvertently magnify these inequities, leading to deeper disparities in healthcare access, diagnosis, and treatment along socioeconomic, racial, gender, and ethnic lines [8–11]. 

Such algorithmic biases can manifest differently across different types of ML. For example, in supervised learning, which uses labelled datasets to classify data or predict outcomes, biases can enter the model through incomplete training data or data that are not representative and lead to inaccurate predictions for diverse populations. In unsupervised learning, biases can take the form of social biases, which encompass a range of prejudicial attitudes rooted in societal factors such as race, gender, and socioeconomic status. These biases may inadvertently emerge during algorithmic analysis of unlabelled data, potentially leading to unfair or discriminatory outcomes, highlighting the importance of addressing them to foster equitable ML practices [12–14]. 

Recently, ML has been acknowledged for improving clinical care, yet less attention has been paid to its applications in population and public health and the potential for biases to arise during model design and development. Our objective was to conduct a scoping review to (1) identify studies that employ ML to address NCDs within the context of population and public health, and (2) to assess any algorithmic bias reporting that may have been exhibited during the design, training, and implementation of ML models, and how model developers mitigated these biases. Examining ML’s role in NCD surveillance informs more effective NCD management and resource allocation, while also addressing algorithmic bias detection to mitigate structural and systemic causes of marginalization in NCD research [12–14]. 

## Methods

This scoping review followed the Preferred Reporting Items for Systematic Reviews and Meta-Analyses Extension for Scoping Reviews (PRISMA-ScR) statement [15]. The protocol for this review was submitted to Open Science Framework (available from osf.io/vkf24/) [16].

### Databases

Due to the multidisciplinary nature of our area of interest, we considered many information sources covering both ML and NCDs. We searched the peer-reviewed, indexed literature using the following databases: *Medline (Ovid)*,* Embase (Ovid)*,* Cochrane Central Register of Controlled Trials and Cochrane Database of Systematic Reviews (Ovid)*,* CINAHL (EBSCOhost)*,* Scopus*,* ACM Digital Library*,* Inspec (Elsevier)*,* and Web of Science’s Science Citation Index*,* Social Sciences Citation Index*,* and Emerging Sources Citation Index*. All languages were included in the search. Commentaries, letters, editorials, conference proceedings were excluded. The databases were searched from 2000 to March 4–7, 2022 (inclusive). The range of publication dates was chosen to identify ML models that use the latest computing approaches and data.

### Search strategy

A health information specialist (CZ) with Library Services, Unity Health Toronto, carried out comprehensive searches using a combination of subject headings and keywords, adapted for each database, for the broad concepts of AI and ML (e.g., artificial neural networks, decision trees, support vector machines) combined using the Boolean operator AND with the following five NCDs: cancers of the lung, trachea, and bronchus, ischemic heart disease, type 2 diabetes, chronic obstructive pulmonary disease, Alzheimer’s disease, and other dementias. Lung, tracheal, and bronchial cancers were chosen specifically as they represent a significant public health burden with high preventability, making them a priority area for exploring the applications of ML. We identified the aforementioned non-communicable diseases (NCDs) as part of the five primary clusters representing the greatest burden of morbidity and mortality caused by NCDs: cancer, cardiovascular disease, diabetes, chronic respiratory diseases, and neurological disorders as specified by the NCD Alliance [17]. Prior to de-duplication, the search yielded 48 701 results. After de-duplication in EndNote, 27 310 references remained. All the search strategies as run are available in Additional file 1 and have also been posted publicly on the Open Science Framework [18]. 

### Eligibility criteria

All studies were required to meet the following eligibility criteria concerning the research focus, at both title/abstract and full-text screening : (1) relevant to population-level health and/or a public health approach; (2) pertain to at least one of the following conditions: cancer of the lung, trachea, and bronchus, ischemic heart disease, type 2 diabetes, chronic obstructive pulmonary disease, Alzheimer’s disease, and other dementias; (3) describe the use of at least one ML model to address a real-world population or public health challenge. There were no language restrictions for the studies reviewed. All study designs were included.

Studies were excluded if: (1) they were not relevant to population-level health and/or a public health approach (i.e., the study focused on individual-level, clinical applications of ML); (2) focus was not any of the conditions mentioned in the inclusion criteria or studies that focused on complications and conditions associated with the condition itself; (3) no-real world data was used; (4) commentaries, letters, editorials, conference proceedings, and dissertations (Table 1).

**Table 1:** Inclusion and exclusion criteria for the study articles

Inclusion Criteria	Exclusion Criteria
(1) Population-wide implications and/or a public health approach, which includes those pertaining to subsets of the general population at a certain point in life-course (e.g., seniors, children).	(1) Did not have a population-wide implication and/or public health approach, this included studies that focused on a population that was defined by one or multiple diseases, studies that focused on domains outside of public health systems or conventional population systems, studies that focused on high-risk groups (e.g., smokers) or in a specialised medical setting (e.g., hospitalized patients), or studies that focused on any subset of the population defined by socio-demographic characteristics other than age, such as ethnicity and sex.
(2) Pertained to at least one of the following conditions: cancer of the lung, trachea, and bronchus, ischemic heart disease, type 2 diabetes, chronic obstructive pulmonary disease, Alzheimer’s disease, and other dementias.	(2) Focus was not any of the conditions mentioned in the inclusion criteria or studies that focused on complications and conditions associated with the condition itself (e.g., diabetic retinopathy).
(3) Described the use of at least one ML model (e.g., artificial neural networks, decision trees, support vector machines) to address a real-world population or public health challenge. There were no language restrictions for the studies reviewed.	(3) No-real world data was used, including general discussions of ML, studies that incorporated data from animal models or in-silico experiments, and proof-of-concept studies.
	(4) Commentaries, letters, editorials, conference proceedings, and dissertations.

### Screening process

DistillerSR was used to manage citations. We trained research assistants to review the citations and test the criteria on 50 randomly selected citations. The training was repeated with randomly selected blocks of 50 citations until inter-rater reliability was met (kappa > 0.9). Reviewers screened the studies via a two-phase process: the title/abstract phase, referred to as first-level screening, and the full-text phase, referred to as second-level screening. The reviewers utilized the eligibility criteria to evaluate and determine the inclusion/exclusion of studies, which were then recorded in DistillerSR.

During first-level screening, two independent reviewers screened titles and abstracts of all imported studies to select studies for final review. If eligibility criteria were fully met, the studies were included. Studies that did not meet at least one inclusion criterion, as agreed upon by the reviewers, were excluded. Conflicts regarding the eligibility of certain studies were resolved through discussion and consensus among the reviewers. If consensus could not be reached, the research associate (CC) decided on inclusion/exclusion. Second-level screening involved reviewing the full-text of all studies that passed the title and abstract screening. This process was performed by a sole reviewer (SB), who excluded any studies that did not meet the same inclusion criteria as the first phase.

### Data collection processing and synthesis

Four independent reviewers extracted data (AP, RR, SB, TV). An Excel data extraction form was developed based on the JBI Manual for Evidence Synthesis [19]. Two reviewers (SB, RR) pilot-tested this form on ten randomly selected studies. The four study team members independently extracted data from all included studies; the data extraction was then vetted by one study team member (SB).

The following data were extracted: author(s), title, journal, year, ML application type(s), intended purpose of ML, study design, intervention (if applicable), results, jurisdiction, data sources, unit(s) of analysis, sample size, demographics, identification of any potential algorithmic bias in the ML model (biases related to gender, sex, ethnicity, socioeconomic status), LMIC transferability, bias mitigation strategies, NCDs targeted, target population and setting, intended users, and impact reported by the author. We also noted if information was unavailable from an article or if any additional sources of algorithmic bias (e.g., age-related bias) were discussed. Narrative syntheses were performed on the extraction categories. The studies were summarized into a table outlining ML applications, ML application aims, jurisdictions, data sources, NCDs studied, considerations of biases and their mitigation (Table 2). The narrative synthesis and synthesis of study characteristics (Table 3) are presented in the Results section.

**Table 2:** Summary of studies that use machine learning to address population and public health challenges surrounding non-communicable diseases (from 2000-present)

Author(s)	Machine Learning Application(s)	Year	Application Aim	Jurisdiction	Data Source	Non-Communicable Disease	Potential Bias; if yes, type?	Mitigation Strategy
Adams et al. [20]	Not Specified	2021	Modelling risk in population	Canada	Longitudinal survey	Cancer of Lung, Trachea, & Bronchus	No	No
Alaa et al. [21]	SVM, RF, DT	2019	Modelling risk in population	United Kingdom	Biomedical Database	CVD	Yes, ethnicity	No
Alexander et al. [22]	NLP	2019	Modelling risk in population	Australia	EMR	Cancer of Lung, Trachea, & Bronchus	No	No
Andy et al. [23]	SVM	2021	Modelling risk in population	United States	Social media textual elements	CVD	No	No
Baechle et al. [24]	NLP	2017	Modelling disease incidence in population	United States (USA)	Biomedical Database	COPD	No	No
Balaji et al. [25]	RF	2022	Modelling disease incidence in population	Germany	EMR	Alzheimer’s & Other Dementias	No	No
Barbieri et al. [26]	Not Specified	2022	Modelling risk in population	New Zealand	Biomedical Database	CVD	Yes, sex	Yes, developed sex-specific deep learning models
Birk et al. [27]	Generalized Linear Model (GLM), Generalized linear mixed model (GLMM), RF, Elastic Net	2021	Modelling risk in population	India	Longitudinal survey	T2D	Yes, not specified	Yes, used GLMM to avoid introducing bias in training set that occurs by assuming responses within families are not correlated
Burnham et al. [28]	SVM	2014	Modelling disease incidence in population	Australia	Longitudinal survey	Alzheimer’s & Other Dementias	No	No
Byeon [29]	RF, DT	2021	Evaluating effectiveness of intervention	Korea	Longitudinal survey	Alzheimer’s & Other Dementias	No	No
Dallora et al. [30]	DT	2020	Modelling risk in population	Sweden	Longitudinal survey	Alzheimer’s & Other Dementias	No	No
Danso et al. [31]	RF, XGBoost	2021	Modelling risk in population	United Kingdom	Longitudinal survey	Alzheimer’s & Other Dementias	No	No
Esmaily et al. [32]	ANN, SVM	2018	Comparison of models/approaches	Iran	Longitudinal survey	T2D	No	No
Esmaeily et al. [33]	RF, DT	2015	Modelling risk in population	Iran	EMR	T2D	No	No
Fazakis et al. [34]	BN, RF, DT, LR	2021	Comparison of models/approaches	United Kingdom	Longitudinal survey	T2D	No	No
Ferdousi et al. [35]	RF, DT, BN, MLP, LR, kNN, SVM-Polykernel & SVM-RBFKernel, Adaboos, Bagging,	2021	Modelling risk in population	Bangladesh	EMR	T2D	No	No
Ford et al. [36]	BN, RF, SVM, ANN	2019	Modelling disease incidence in population	United Kingdom	Biomedical Database	Alzheimer’s & Other Dementias	No	No
Gholipour et al. [37]	ANN, Multiple Regression	2018	Comparison of models/approaches	Iran	EMR	T2D	No	No
Goldman et al. [38]	ANN	2021	Comparison of models/approaches	United States	Longitudinal survey	CVD	No	No
Haneef et al. [39]	LDA, LR, Flexible Discriminant Analysis, DT, Boosted C5, XGBoost	2021	Modelling disease incidence in population	France	Longitudinal survey	T2D	Yes, age-related bias	Yes, random resampling to balance the training and data set (lack of older population in dataset)
Hu et al. [40]	RF, XGBoost, BN, LR	2021	Modelling disease incidence in population	China	Longitudinal survey	Alzheimer’s & Other Dementias	No	No
Jia et al. [41]	Markov Modelling	2020	Modelling risk in population	United States	Longitudinal survey	Alzheimer’s & Other Dementias	No	No
Kamis et al. [42]	RF, DT, SVM	2021	Comparison of models/approaches	United States	Laboratory Data	Cancer of Lung, Trachea, & Bronchus	No	No
Kim et al. [43]	DNN	2021	Comparison of models/approaches	South Korea	Longitudinal survey	T2D	No	No
Kim et al. [44]	DNN, RF, Adaboost, MLP, BN, SVM	2021	Comparison of models/approaches	South Korea	Longitudinal survey	Alzheimer’s & Other Dementias	No	No
Kim et al. [45]	ANN	2017	Modelling risk in population	South Korea	Longitudinal survey	CVD	No	No
Lam et al. [46]	Unsupervised Learning	2021	Modelling risk in population	United Kingdom	Wearable Sensor	T2D	No	No
Liao et al. [47]	RF, GB, Bagging	2019	Modelling disease incidence in population	United States	Administrative Claims	T2D	No	No
Lim et al. [48]	RF, MLP, SVM	2021	Modelling risk in population	South Korea	Administrative Claims	Alzheimer’s & Other Dementias	No	No
Lim et al. [49]	ANN, Deep Belief Network (DBN)	2018	Modelling risk in population	South Korea	Longitudinal survey	Cancer of Lung, Trachea, & Bronchus	Yes, ethnicity	No
Liu et al. [50]	Ensemble Methods (Voting and Stacking)	2019	Modelling risk in population	China	Biomedical Database	T2D	No	No
Liu et al. [51]	ANN, DT, LR	2019	Modelling risk in population	China	EMR	T2D	No	No
Mani et al. [52]	BN, LR, kNN, RF, SVM	2012	Comparison of models/approaches	United States	EMR	T2D	No	No
Mar et al. [53]	RF	2022	Modelling disease incidence in population	Spain	Biomedical Database	Alzheimer’s & Other Dementias	Yes, age-related bias	No
Masih et al. [54]	MLP	2021	Comparison of models/approaches	United States	Longitudinal survey	CVD	No	No
Moon et al. [55]	Not Specified	2021	Modelling risk in population	South Korea	Longitudinal survey	T2D	No	No
Nayak et al. [56]	Local Linear Wavelet Neural Network (LLWN), Structured Singular Value (SSV), Simplex Method based Social Spider Optimization (SMSSO)	2022	Evaluating effectiveness of intervention	India	Biomedical Database	T2D	No	No
Neumann et al. [57]	Not Specified	2022	Modelling risk in population	Australia	Longitudinal survey	CVD	No	No
Ooka et al. [58]	RF	2021	Modelling risk in population	Japan	EMR	T2D	Yes, not specified	No
Owusu et al. [59]	BN, LR, MLP, SVM, DT	2017	Comparison of models/approaches	United Kingdom	Biomedical Database	T2D	Yes, sex	Combined human expertise with machine power to represent best strategy to test hypothesis on potential disease predictors
Park et al. [60]	Not Specified	2020	Modelling disease incidence in population	South Korea	Administrative Claims	Alzheimer’s & Other Dementias	No	No
Park et al. [61]	ANN, Multiple Regression (MRM), Sequential Neural Network (SNN)	2001	Modelling disease incidence in population	United States	Administrative Claims	T2D	No	No
Patil et al. [62]	SVM	2022	Modelling disease incidence in population	India	EMR	T2D	No	No
Pekkala et al. [63]	SVM	2016	Modelling disease incidence in population	Finland	Longitudinal survey	Alzheimer’s & Other Dementias	No	No
Piko et al. [64]	LR	2020	Modelling risk in population	Hungary	Longitudinal survey	T2D	Yes, sex	Data of each sex was analysed separately; saw no prominent differences
Priyanga et al. [65]	SVM, kNN, ANN, BN	2020	Comparison of models/approaches	India	EMR	CVD	No	No
Ravaut et al. [66]	DT	2021	Modelling risk in population	Canada	EMR	T2D	Yes, unspecified	No
Razavian et al. [67]	Not Specified	2015	Modelling risk in population	United States	Administrative Claims	T2D	No	No
Rehman et al. [68]	RF	2021	Modelling disease incidence in population	United States	Search-Engine Queries	COPD	No	No
Shangguan et al. [51]	BN	2021	Modelling risk in population	China	Longitudinal survey	COPD	Yes, sex and socioeconomic status	Adjusted for sex and income as risk factors of COPD
Su et al. [69]	ANN	2021	Modelling disease incidence in population	Taiwan	Administrative Claims	T2D	No	No
Su et al. [70]	RF	2020	Modelling disease incidence in population	China	EMR	CVD	No	No
Syed et al. [71]	Synthetic Minority Over-sampling Technique (SMOTE)	2020	Modelling risk in population	Saudi Arabia	Longitudinal survey	T2D	No	No
Uddin et al. [72]	SVM, RF, kNN, ANN, LR	2022	Modelling risk in population	Australia	Administrative Claims	T2D	Yes, not specified	No
Wang et al. [73]	SVM	2021	Modelling risk in population	China	EMR	CVD	No	No
Wang et al. [74]	SVM, RF	2021	Modelling risk in population	China	Longitudinal survey	T2D	No	No
Wu et al. [75]	Regularized Logistic Regression (r-LR), SVM, RF, Super Learner (SL)	2022	Modelling risk in population	China	Longitudinal survey	Alzheimer’s & Other Dementias	No	No
Xie et al. [76]	BN, DT, LR, ANN, RF, SVM	2019	Modelling risk in population	United States	Cellular Data	T2D	No	No
Xiong et al. [77]	MLP, AdaBoost, RF, SVM, GB	2019	Comparison of models/approaches	China	EMR	T2D	Yes, ethnicity and socioeconomic status (SES)	No
Yang et al. [78]	LDA, SVM, RF	2020	Modelling risk in population	China	Longitudinal survey	T2D	Yes, not specified	Yes, not specified
Yeh et al. [79]	DNN	2021	Modelling disease incidence in population	Taiwan	Administrative Claims	Cancer of Lung, Trachea, & Bronchus	Yes, ethnicity	No
Yun et al. [80]	Deep Learning (DL)	2022	Modelling risk in population	United Kingdom	Biomedical Database	T2D	No	No
Zafari et al. [81]	Multilayer Neural Networks (MLNN), Extreme Gradient Boosting (XGB)	2022	Modelling disease incidence in population	Canada	EMR	COPD	No	No
Zhang et al. [82]	RF, ANN, DT	2020	Comparison of models/approaches	Australia	Longitudinal survey	T2D	Yes, sex and ethnicity	No
Zheng et al. [83]	NLP, RF	2016	Modelling disease incidence in population	United States	EMR	T2D	No	No

*Abbreviations*: *ANN* artificial neural network, *BN* naïve bayesian network, *COPD* chronic obstructive pulmonary disease, *CVD* cardiovascular disease, *DT* Decision Tree, *DNN* deep neural network, *EMR* electronic medical record, *GB* gradient boosting, *kNN* k-Nearest Neighbour, *LDA* linear discriminant analysis, *LR* logistic regression, *MLP* multilayer perceptron, *NLP* natural language processing, *RF* random forest, *SVM* support vector machine, *T2D* type 2 diabetes

**Table 3:** Characteristics of included studies

	Characteristic	Frequency	%
**Years of Publication**	2005–2010	1	1.54
	2011–2016	5	7.69
	2017–2023	59	90.77
**Jurisdictions**	Bangladesh	1	1.54
	Canada*	3	4.62
	China	10	15.38
	Finland*	1	1.54
	France*	1	1.54
	Germany*	1	1.54
	Hungary*	1	1.54
	India	4	6.15
	Iran	3	4.62
	Japan*	1	1.54
	New Zealand*	1	1.54
	Saudi Arabia*	1	1.54
	South Korea*	8	12.31
	Spain*	1	1.54
	Sweden*	1	1.54
	Taiwan*	2	3.08
	United Kingdom*	7	10.77
	United States*	13	20.00
**Application Aims**	Modelling Risk in Population	32	61.54
	Modelling Disease Incidence in Population	18	34.62
	Evaluating Effectiveness of Intervention	2	3.85
	Comparison of Models/Approaches	13	20.00
**Data Sources**	Longitudinal Survey Data	27	41.54
	Biomedical databases	9	13.85
	Electronic medical records	16	24.62
	Social media textual elements	1	1.54
	Administrative claims	8	12.31
	Laboratory data	1	1.54
	Cellular data	1	1.54
	Search engine queries	1	1.54
**Non-communicable diseases**	Type 2 diabetes	32	49.23
	Alzheimer’s & other dementias	14	21.54
	Cardiovascular disease	10	15.38
	Chronic obstructive pulmonary disease	4	6.15
	Cancer or the lung, trachea, and bronchus	5	7.69
**Major technical approaches**	Support vector Machine	21	32.31
	Multilayer perceptron	6	9.23
	Random forest	27	41.54
**Bias considerations**	No consideration	49	75.38
	Ethnicity-related bias	5	7.69
	Sex-related bias	5	7.69
	Age-related bias	2	3.85
	Socioeconomic status	2	3.85
	Not specified bias	5	7.69
**Implementation of bias mitigation strategies**	Yes	7	10.76
	No	58	89.23

Numerous ML approaches were used simultaneously in certain studies

More than one bias was considered in certain studies

*High-income economy (i.e., for 2023, with a gross national income (GNI) per capita of $13 205 or more)

## Results

### Study selection

**Fig. 1:**
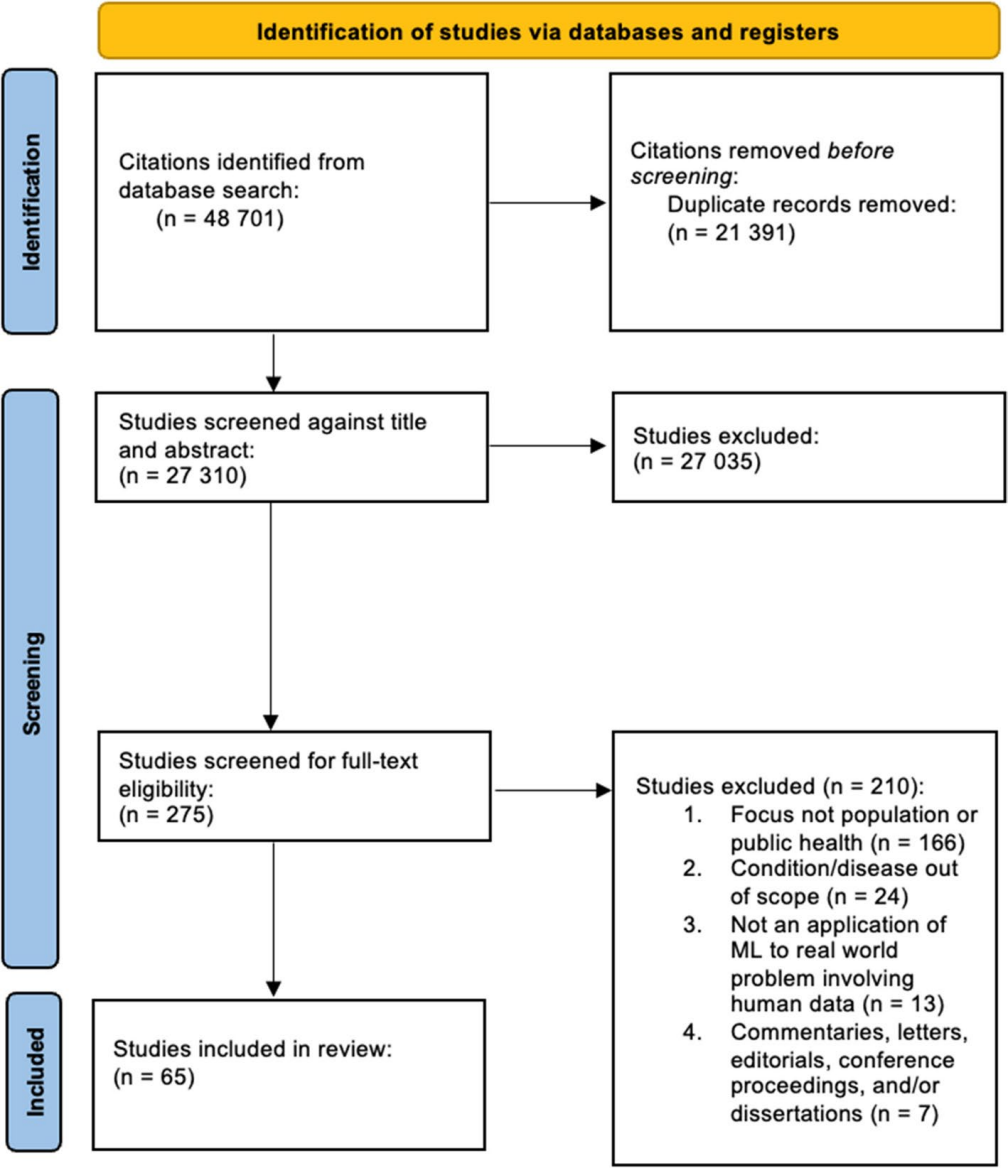
PRISMA-ScR flow diagram

Our initial search yielded 27 310 citations. Following title/abstract screening, 275 abstracts remained. Following full-text screening by SB, 65 articles met eligibility criteria and were included in the final review (Fig. 1).

### Publication and study characteristics

**Fig. 2:**
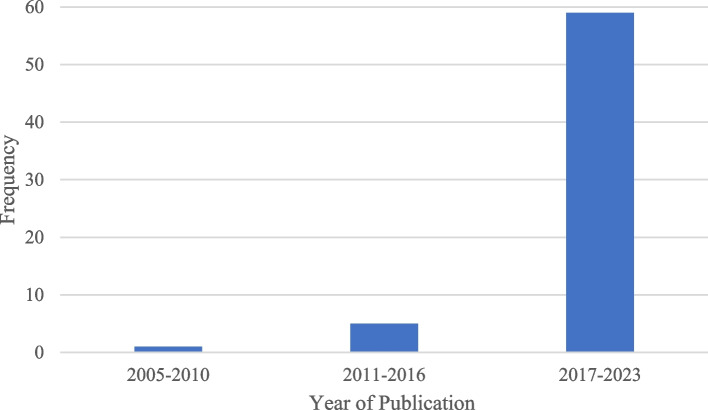
Distribution of included studies by year of publication

Table 2 presents a summary of the data extracted from each included study. Most of the studies (*n* = 59, 90.77%) were published between 2017 and 2023; five studies (*n* = 5, 7.69%) were published between 2011 and 2016; and one study (*n* = 1, 1.54%) was published between 2005 and 2010 (Fig. 2).

Table 3 presents a synthesis of the characteristics of the included studies and the frequency with which each of the following subcategories was reported.

### Application aims

Studies could be classified as either comparing ML models/approaches (*n* = 13, 20.00%) [33, 34, 37, 38, 42–44, 52, 54, 59, 65, 77, 82] or using disease modelling for population-health related outputs (*n* = 52, 80.00%) [20–32, 35, 36, 39–41, 45–51, 53, 55–58, 60–64, 66–76, 78–81, 83, 84]. The modelling of NCDs included measuring incidence in the population (*n* = 18, 34.62%) [24, 25, 28, 36, 39, 40, 47, 53, 60–63, 68–70, 79, 81, 83], measuring risk in the population (*n* = 32, 61.54%) [20–23, 26, 27, 30–32, 35, 41, 45, 46, 48–51, 55, 57, 58, 64, 66, 67, 71–76, 78, 80, 84] and evaluating the effectiveness of an inervention on outcomes as defined by study authors (*n* = 2, 3.85%) [29, 56].

### Data sources

Data sources used by the studies included longitudinal survey data (*n* = 27, 41.54%) [20, 27–31, 33, 34, 38–41, 43–45, 49, 51, 54, 55, 57, 63, 64, 71, 74, 75, 78, 82], biomedical databases (*n* = 9, 13.85%) [21, 24, 26, 36, 50, 53, 56, 59, 80], electronic medical records (*n* = 16, 24.62%) [22, 25, 32, 35, 37, 52, 58, 62, 65, 66, 70, 73, 77, 81, 83, 84], social media textual elements (*n* = 1, 1.54%) [23], administrative claims (*n* = 8, 12.31%) [47, 48, 60, 61, 67, 69, 72, 79, 82], laboratory data (*n* = 1, 1.54%) [42], cellular data (*n* = 1, 1.54%) [76], search-engine queries (*n* = 1, 1.54%) [68], and wearable sensors (*n* = 1, 1.54%) [46] .

### Non-communicable diseases targeted

Almost 50% (*n* = 32, 49.23%) [27, 32–35, 37, 39, 43, 46, 47, 50, 52, 55, 56, 58, 59, 61, 62, 64, 66, 67, 69, 71, 72, 74, 76–78, 80, 82–84] of included studies focused on type 2 diabetes. Almost a quarter examined Alzheimer’s and other dementias (*n* = 14, 21.54%) [25, 28–31, 36, 40, 41, 44, 48, 53, 60, 63, 75]. Around 30% of included studies focused on cardiovascular and respiratory diseases (*n* = 19, 29.23%) [20–24, 26, 38, 42, 45, 49, 51, 54, 57, 65, 68, 70, 73, 79, 81], spanning specifically ischemic heart disease (*n* = 10, 52.63%) [21, 23, 26, 38, 45, 54, 57, 65, 70, 73], chronic obstructive pulmonary disease (*n* = 4, 21.05%) [24, 51, 68, 81], and cancer of the lung, trachea, and bronchus (*n* = 5, 26.32%) [20, 22, 42, 49, 79].

### Technical approaches

The approach most employed within the applications studied was supervised learning, aimed at resolving problems of, or completing tasks involving classification and/or regression. Included in this paradigm are approaches involving constructs such as decision trees, ensembles (in turn including bagging, boosting, and random forest constructs), algorithms such as k-nearest neighbor, and systems such as artificial neural and naïve Bayesian networks. In terms of specific technologies, some algorithms as well as constructs employed within the studies considered were support vector machine (*n* = 21, 32.31%) [21, 23, 28, 33, 35, 36, 42, 44, 48, 52, 59, 62, 63, 65, 72–78], multilayer perceptron (*n* = 6, 9.23%) [35, 44, 48, 54, 59, 77], random forest (*n* = 27, 41.54%) [21, 25, 27, 29, 31, 32, 34–36, 40, 42, 44, 47, 48, 52, 53, 58, 68, 70, 72, 74–78, 82, 83]. Numerous ML approachs were used simultaneously in certain studies.

### Consideration of bias and its mitigation

Although all the reviewed articles recommended applying their ML models in their specific NCD contexts, less than one-third (*n* = 16, 24.62%) [21, 26, 27, 39, 49, 51, 53, 58, 59, 64, 66, 72, 77–79, 82] addressed the possibility of algorithmic bias that may arise from the implementation of their ML models. Of those studies mentioning bias potential, only 7 (7 out of 16, 43.75%) [26, 27, 39, 51, 59, 64, 78] outlined practical steps taken to mitigate bias. For the most part, those methods concerned mitigating sex-related bias and affected model design.

### Countries and other regional divisions represented

Areas from which samples were drawn included Australia (*n* = 5, 7.69%) [22, 28, 57, 72, 82], Bangladesh (*n* = 1, 1.54%) [35], Canada (*n* = 3, 4.62%) [20, 66, 81], China (*n* = 10, 15.38%) [40, 50, 51, 70, 73–75, 77, 78, 84], Finland (*n* = 1, 1.54%) [63], France (*n* = 1, 1.54%) [39], Germany (*n* = 1, 1.54%) [25], Hungary (*n* = 1, 1.54%) [64], India (*n* = 4, 6.15%) [27, 56, 62, 65], Iran (*n* = 3, 4.62%) [32, 33, 37], Japan (*n* = 1, 1.54%) [58], New Zealand (*n* = 1, 1.54%) [26], Saudi Arabia (*n* = 1, 1.54%) [71], South Korea (*n* = 8, 12.31) [29, 43–45, 48, 49, 55, 60], Spain (*n* = 1, 1.54%) [53], Sweden (*n* = 1, 1.54%) [30], Taiwan (*n* = 2, 3.08%) [69, 79], the United Kingdom (*n* = 7, 10.77%) [21, 31, 34, 36, 46, 59, 80], and the United States (*n* = 13, 20.00%) [23, 24, 38, 41, 42, 47, 52, 54, 61, 67, 68, 76, 83] .

Most studies used datasets drawn from areas defined by the World Bank as high-income economies (i.e. for 2023, those with a gross national income (GNI) per capita of $13 205 or more) (*n*= 15/19 total countries; 78.95%) [85]. The remainder originated from countries classified as lower-middle-income (i.e. those with a GNI per capita of $1 086 to $4 255 [85], here specifically Bangladesh, India, and Iran) or upper-middle income (i.e. those with a GNI per capita of $4 256 to $13 204, here China) [85]. 

## Discussion

### Summary

In summary, we identified 65 peer-reviewed studies published since 2005 that applied ML methods to evaluate NCDs using a population health lens. Only 65 out of 27 310 references were eligible for our study, illustrating the lack of studies that comment on ML applications in population and public health, specifically concerning NCDs. The initial large reference yield may have been due to the novelty of ML and, after the onset of COVID-19, the increasing interest into population and public health. Although the literature adequately addressed types of data sources, to truly engage with issues of health equity, more work must be done to address algorithmic biases in ML which leaves a gap for researchers to explore.

### Study selection and methodological considerations

We employed a rigorous selection process to determine which research studies would be included in our analysis. This process involved applying specific criteria, which ultimately led to the exclusion of certain studies. The reasons behind the exclusion of these studies are comprehensively outlined in Table 1. Some studies were later eliminated from consideration for various reasons, despite initially meeting our inclusion criteria. Firstly, some studies’ samples were obtained in ways that were not representative of the broader population. For instance, while one study by Muro et al. (2021) geared at identifying predictors of COPD diagnosis using data from many of the same individuals’ annual medical check-up information across 21 years, these individuals were all employees of Hitachi, Ltd. [86], which could have systematically influenced some aspect of the data collection. Secondly, other studies’ objectives, upon full-text examination, differed from what we identified during our initial screening. For example, one study titled “Predicting Lung Cancer in the United States: A Multiple Model Examination of Public Health Factors” appeared initially to model disease incidence and specify risk factors but ultimately focused on which emitted compounds are most harmful, and how population health can be improved by initiatives geared at transitioning the USA from non-renewable to renewable energy sources [42]. Because this study did not ultimately overview participants’ data (i.e., no sample size was mentioned), it did not meet our inclusion criteria.

### Future directions in NCD research and public health interventions

The diseases evaluated in this review are representative of the global burden of mortality from NCDs, emphasizing diabetes, ischemic heart disease, cancers, and chronic respiratory diseases [87]. Study-focus distribution was also indicative of respective disease-category burdens. For instance, 49.23% of studies centered on T2D. Mortality from diabetes is increasing at a higher rate than other NCDs [88]. While overall NCD age-standardized mortality rates decreased by 22% globally between 2000 and 2019 for those between the ages of 30 and 70, diabetes age-standardized mortality for the same group increased by 3% worldwide [88]. At the same time, there has been a notable 13% increase in mortality rates attributable to T2D in LMICs [88]. The burden of disease is of particular relevance to LMICs, where there is already a high burden of infectious diseases. However, this was not reflected in the ML applications examined across this review. In contrast to the substantial 48.48% of studies that predominantly focused on Type 2 Diabetes (T2D), a significantly smaller fraction of studies (around 16%) tackled cardiovascular diseases (CVDs). Although T2D mortality is increasing where CVDs’ is decreasing, T2D is still directly responsible for fewer deaths: approximately two million relative to 17.9 million from CVDs [87]. As such, CVD-centered ML applications in health may be useful to prioritize, considering the vast spectrum of conditions which could be categorized as CVDs [89]. 

### Advancements in ML approaches

From a technical standpoint, supervised learning was the most popular algorithm found in our search. Unsupervised learning was also employed in some studies, such as in Lam et al. (2021) [46]. These approaches can uncover patterns in data and identify subpopulations, making them particularly useful for exploratory analysis. Specifically, Lam et al. demonstrated the potential of continuous or periodic self-monitoring for early detection and screening of disease progression among subpopulations at risk of T2D, particularly those in a prediabetic state [46]. Principal component analysis (PCA), a popular dimensionality reduction technique, was used by Kim et al. (2021) [44] to predict not only future dementia patients but also other types of diseases using data that include limited input variables, making it useful in places with limited access to resources. The findings suggest that PCA can serve as a cost-effective tool for predicting future cases of dementia and other diseases, even with limited input variables [44]. Natural language processing (NLP) and text mining techniques were used by Alexander et al. (2020), Zheng et al. (2016), and Baechle et al. (2017) to extract information from electronic health records to identify disease patterns and risk factors [22, 24, 83]. The results of these studies demonstrate the potential of NLP and text mining techniques in extracting population health data from large-scale electronic health records, which could contribute to developing more targeted public health interventions.

Risk modelling was the most popular application of ML. Ravaut et al. (2021) and Barbieri et al. (2022) established a machine-learning model at a population level that accurately predicts the onset of T2D and CVD using administrative health data up to 5 years in advance [26, 66]. The studies suggested that using ML and administrative health data can create effective population health planning tools to differentiate high-risk from low-risk populations for diabetes. This can assist in directing investments and interventions toward preventing NCDs and could also aid in mitigating individual-level complications.

### Transferability of ML applications to resource-limited settings

There is a notable disparity in the frequency of ML applications between high-income and low- and middle-income countries. Populations classified as “low-income” by the World Bank were not included in the studies considered within this review. The application of ML models in jurisdictions that lack robust health records may be limited as these approaches rely on large-scale data sets to learn patterns and make predictions [90]. However, one study led by researchers from the United States explored the use of several ML techniques as a lower-cost alternative to prediabetes screening in resource-limited settings [27]. The authors used survey data from an FFQ completed by individuals from a rural region of Hyderabad, India, to calculate each participant’s Global Diet Quality Score (GDQS) and predict their risk for T2D development. The global applicability of the GDQS combined with ML techniques served as a low-cost, easy-to-use method for identifying populations at high risk of developing diabetes, bypassing the need to screen all individuals using laboratory-based tests [27]. An example of a promising data source is social media textual elements, such as Facebook posts, to help predict the risk of an NCD. In a study by Andy et al., the discriminatory ability of social media posts to predict the 10-year risk of CVD was compared to that of pooled cohort risk equations [23]. The study results present a novel outlook for utilizing new and emerging digital data sources to identify potential risk factors by analyzing information recorded over several time points [23]. Accessing the rapidly generated data on social media platforms (e.g., posts) from consenting individuals offers an opportunity to collect and analyze unscripted information that can differ from the standard survey assessments.

### Geographical representation and generalizability of data

Within countries, there was inadequate representation of different regions. For instance, one study collected a diverse set of demographic variables (i.e., participant diet and level of cultural participation) [74] but was limited in its generalizability to a broader Chinese population because it focused on data collected solely from the Shanxi region. Similarly, another study featuring a sample from China focused on Nanjing. While focusing on regional samples provides insight into specific subpopulations’ health in China, it also highlights a shortfall in data on other subpopulations. This could be because population health data and public health initiatives appear to be provincially governed.

This trend appears to be applicable to several nations. Other studies focused on regions such as East Azerbaijan [37] and Mashhad, Iran [33], and eight cities in Tamil Nadu, India [65]. One study aimed at predicting participation in a cognitive health promotion program among older adults in Seoul, South Korea, who had not been diagnosed with MND. This study focused on correlating intent to participate with various demographic factors such as level of education, smoking status, and cohabitation status [29]. The authors recognized the complex biopsychosocial nature of cognitive health and employed a methodology that equalized city-level representation, such as stratified clustered sampling of all 25 districts in Seoul [29]. Since most of South Korea’s population resides in urban regions [91], generalizability to the entire population may not be as significant.

### Addressing algorithmic bias in ML

With respect to algorithmic bias, there was an overall lack of discussion on identifying, defining, and mitigating bias in population health settings. Chen et al. (2021) explored how the potential for ML to exacerbate existing health disparities, especially during model development, is a concern that requires more attention [92]. The article stresses the importance of health data in ML models and notes how collected data can be biased, with a larger portion of the dataset leaning towards a specific biological sex or gender-identity, for example [92]. In this case, the model cannot be initialized due to imbalanced baseline representation [92]. A study by Barbieri et al. attempts to mitigate sex-related bias to detect CVD by developing sex-specific ML models, emphasising the improved calibration and discrimination enabling 5-year risk prediction [26]. Yet, it also emphasizes the need to further explore these models in countries with larger administrative health datasets [26]. However, even with larger datasets, algorithmic biases are still present [92]. People made vulnerable by social and economic policies, including transgender and gender-nonconforming individuals, undocumented immigrants, and racialized populations are often underrepresented, misrepresented, or missing from collected health data [92]. Demographic data collected in countries such as Canada and France, where race and ethnicity are not recorded in their nationalized health databases, makes race-based disparities extremely difficult to explore [92]. Ultimately, representative data collection is important in ensuring that datasets reflect the public population [92]. Moreover, the lack of reporting on bias in studies on population health must be addressed if ML is regarded as being able to revolutionize global healthcare systems [92, 93]. 

### Strengths and limitations

This review is novel in examining how ML has been applied to population and public health by a range of applications such as prediction, surveillance, and evaluating the effectiveness of interventions. Notably, we identified potential algorithmic biases and mitigation strategies. This review has several limitations. Firstly, a grey literature search was not conducted, thus possibly introducing selection bias. Next, we did not perform duplicate screening during the full-text screening phase to adhere to project timelines and resources. Similarly, while data extraction was vetted by the lead author, it was not conducted in duplicate. This approach may introduce the potential for bias, particularly in areas requiring subjective judgment, such as interpreting the inclusion criteria and determining which biases were discussed in the studies and how they were mitigated. Although our reviewer (SB) had extensive experience in the topic area, the lack of duplicate screening may affect the reliability of our findings. Additionally, although we did not place any restrictions on language, non-English articles were translated via Google Translate which is susceptible to some level of error. Finally, the terms *population health* and *machine learning* are not universally defined. Although we tried to encompass subtypes of machine learning in our search strategy, we may have excluded articles that could have relevance to the field. Along the same lines, increased recognition of the complexities of NCD-NCD interplays and, more broadly, the finer aspects of keyword delineation will characterize future work. This includes the preferred terminology used by structurally disadvantaged communities to describe their experiences and the terminological conventions used to discuss ML applications in health in languages other than English. Finally, this review acknowledges the limitation of focusing solely on lung, tracheal, and bronchial cancers. While this allowed for in-depth analysis within this specific scope, future research incorporating a wider range of cancer types is necessary to gain a more comprehensive understanding of ML applications in oncology.

## Conclusion

This review provides an overview of current ML applications as well as the potential for bias and bias mitigation strategies. This was the first scoping review focused on ML applications for studying NCDs. LMIC transferability of such ML models was not discussed much, leaving a gap for researchers to investigate data transparency methods, such as making codes and protocols open source. As the field of ML continues to evolve, there will be ample opportunity to capitalize upon the use of technology to improve population health (e.g., identifying high-risk subgroups); we hope our results will help to guide future research, such as the development of guidelines for the equitable use of machine learning.

## Supplementary Information

Additional file 1.

## Data Availability

The datasets used and/or analysed during the current study are available from the corresponding author upon reasonable request.
