# Supplementary material for: Bias in machine learning applications to address non-communicable diseases at a population-level: a scoping review
Source: BMC Public Health. 2024 Dec 28;24:3599. doi: 10.1186/s12889-024-21081-9 (PMC11682638; doi:10.1186/s12889-024-21081-9)
Supplement: Supplementary file 1 — Additional file 1. [file 12889_2024_21081_MOESM1_ESM.docx]

**Additional File 1:**

**Literature Search Strategies for: Bias in machine learning applications to address non-communicable diseases at a population-level: a scoping review**

Searches designed in collaboration with the team and conducted on March 4-7, 2022 by Carolyn Ziegler, Information Specialist, Library Services, Unity Health Toronto

**Summary of Results per Database**

| **Database** | **Number of Results** |
| --- | --- |
| Medline (Ovid) | 10,055 |
| Embase (Ovid) | 16,288 |
| Cochrane Central Register of Controlled Trials and  Cochrane Database of Systematic Reviews (EBM Reviews, Ovid, searched simultaneously | 578 |
| CINAHL (Ebscohost) | 2,480 |
| Web of Science selected databases, searched simultaneously:  Science Citation Index Expanded  Social Sciences Citation Index  Emerging Sources Citation Index | 9,957 |
| Scopus | 5,878 |
| ACM Digital Library (Association for Computing Machinery) | 66 |
| Inspec (Engineering Village/Elsevier) | 3,399 |
| **Total Number of Results** | **48,701** |
| **Total Number of Results after De-duplication in EndNote** | **27,310** |

**Search Strategies:**

**Ovid MEDLINE: Epub Ahead of Print, In-Process & Other Non-Indexed Citations, Ovid MEDLINE® Daily and Ovid MEDLINE® <1946-Present>**

1 artificial intelligence/ or machine learning/ or deep learning/ or supervised machine learning/ or support vector machine/ or unsupervised machine learning/ 68003

2 computer heuristics/ or natural language processing/ or neural networks, computer/ 42116

3 artificial intelligence.tw,kf. 20467

4 Machine learning.tw,kf. 58409

5 neural network*.tw,kf. 72790

6 deep learning.tw,kf. 27619

7 supervised learning.tw,kf. 3840

8 unsupervised learning.tw,kf. 1950

9 deep architecture*.tw,kf. 242

10 computational intelligence.tw,kf. 374

11 computer reasoning.tw,kf. 7

12 machine intelligence.tw,kf. 203

13 support vector machine*.tw,kf. 20464

14 support vector network*.tw,kf. 7

15 natural language processing.tw,kf. 5389

16 data driven algorithm*.tw,kf. 143

17 perceptron.tw,kf. 3136

18 random forest*.tw,kf. 14523

19 (ensemble learning or reinforcement learning).tw,kf. 5804

20 AI.ti. 3277

21 or/1-20 [Machine Learning] 198528

22 exp Respiratory Tract Neoplasms/ 315233

23 (lung* adj3 (cancer* or neoplas* or adenocarcinoma* or carcinoma* or malignan* or tumour* or tumor* or blastoma* or metast*)).tw,kf. 267163

24 (pulmonary adj3 (cancer* or neoplas* or adenocarcinoma* or carcinoma* or malignan* or tumour* or tumor* or blastoma* or metast*)).tw,kf. 30486

25 (bronch* adj3 (cancer* or neoplas* or adenocarcinoma* or carcinoma* or malignan* or tumour* or tumor* or blastoma* or metast*)).tw,kf. 21929

26 (alveolar adj3 (cancer* or neoplas* or adenocarcinoma* or carcinoma* or malignan* or tumour* or tumor* or blastoma* or metast*)).tw,kf. 2256

27 (trachea* adj3 (cancer* or neoplas* or adenocarcinoma* or carcinoma* or malignan* or tumour* or tumor* or blastoma* or metast*)).tw,kf. 2659

28 (pleural adj3 (cancer* or neoplas* or adenocarcinoma* or carcinoma* or malignan* or tumour* or tumor* or blastoma* or metast*)).tw,kf. 12024

29 (Malignant Mesothelioma or Multiple Pulmonary Nodules or Pancoast Syndrome or pancoast tumor* or pancoast tumour*).tw,kf. 6124

30 or/22-29 [cancers of the lung, trachea, bronchus] 426506

31 exp Myocardial Ischemia/ 454711

32 (Myocardial Ischemia* or Myocardial Ischaemia or ischemic heart disease* or ischaemic heart disease* or Acute Coronary Syndrome* or angina* or angor pectori* or myocardial preinfarction syndrome* or stenocardia* or coronary artery insufficiency or coronary artery occlusive disease* or coronary heart disease* or coronary insufficiency or coronary occlusive disease* or ischaemic cardiac disease* or ischaemic cardial disease* or ischaemic cardiopathy or ischemic cardiac disease* or ischemic cardial disease* or ischemic cardiopathy or Coronary Disease* or Coronary Aneurysm* or Coronary Artery Disease* or Coronary Occlusion or Coronary Stenosis or Coronary Restenosis or Coronary Thrombosis or Coronary Vasospasm or Myocardial Infarct* or stemi or cardiovascular stroke* or heart attack*).tw,kf. 449766

33 31 or 32 [ischemic heart disease] 601919

34 Diabetes Mellitus, Type 2/ 153081

35 (type* adj3 two adj3 diabet*).tw,kf. 1216

36 (type* adj3 "2" adj3 diabet*).tw,kf. 160912

37 (type* adj3 "II" adj3 diabet*).tw,kf. 12157

38 (adult* adj3 onset adj3 diabet*).tw,kf. 945

39 (Matur* adj3 onset adj3 diabet*).tw,kf. 2333

40 (slow adj3 onset adj3 diabet*).tw,kf. 29

41 (Ketosis resistant adj3 diabet*).tw,kf. 27

42 (stable adj3 diabet*).tw,kf. 808

43 (Non insulin adj3 dependent adj3 diabet*).tw,kf. 10566

44 (Noninsulin adj3 dependent adj3 diabet*).tw,kf. 1390

45 NIDDM.tw,kf. 6970

46 MODY.tw,kf. 1481

47 "diabet*".m_titl. 378239

48 or/34-47 [Type 2 Diabetes] 459396

49 exp Pulmonary Disease, Chronic Obstructive/ 62295

50 (chronic obstructive lung disease or chronic airflow obstruction or chronic airway obstruction or chronic obstructive bronchopulmonary disease or chronic obstructive lung disorder or chronic obstructive pulmonary disease or chronic obstructive pulmonary disorder or chronic obstructive respiratory disease or chronic pulmonary obstructive disease or chronic pulmonary obstructive disorder or copd or lung chronic obstructive disease or obstructive chronic lung disease or obstructive chronic pulmonary disease or chronic bronchitis or emphysema).tw,kf. 104651

51 49 or 50 [COPD] 115074

52 exp Dementia/ 187496

53 (Dementia* or demented or amentia* or alzheimer* or Primary Progressive aphasia or mesulam syndrome or Creutzfeldt Jakob Syndrome or creutzfeldt jakob disease or CADASIL or Frontotemporal Lobar Degeneration or pick* disease or lobar atroph* or Huntington* Disease or huntington* chorea or Kluver Bucy Syndrome or Lewy Body Disease* or Lewy Body Disorder*).tw,kf. 276081

54 52 or 53 [Alzheimer's disease and other dementias] 300263

55 21 and (30 or 33 or 48 or 51 or 54) 10733

56 limit 55 to (comment or editorial or letter) 200

57 55 not 56 10533

58 57 not (exp animals/ not humans.sh.) 10353

59 limit 58 to yr="2000 -Current" 10055

**Embase Classic+Embase <1947 to 2022 March 03>**

1 exp machine learning/ 296335

2 exp artificial intelligence/ 58023

3 natural language processing/ 7423

4 (artificial intelligence or Machine learning or neural network* or deep learning or supervised learning or unsupervised learning or deep architecture* or computational intelligence or computer reasoning or machine intelligence or support vector machine* or support vector network* or natural language processing or data driven algorithm* or perceptron or random forest* or ensemble learning or reinforcement learning).tw,kf. 204248

5 AI.m_titl. 4385

6 1 or 2 or 3 or 4 or 5 374773

7 exp respiratory tract cancer/ 422085

8 (lung* adj3 (cancer* or neoplas* or adenocarcinoma* or carcinoma* or malignan* or tumour* or tumor* or blastoma* or metast*)).tw,kf. 401120

9 (pulmonary adj3 (cancer* or neoplas* or adenocarcinoma* or carcinoma* or malignan* or tumour* or tumor* or blastoma* or metast*)).tw,kf. 46217

10 (bronch* adj3 (cancer* or neoplas* or adenocarcinoma* or carcinoma* or malignan* or tumour* or tumor* or blastoma* or metast*)).tw,kf. 32870

11 (alveolar adj3 (cancer* or neoplas* or adenocarcinoma* or carcinoma* or malignan* or tumour* or tumor* or blastoma* or metast*)).tw,kf. 3314

12 (trachea* adj3 (cancer* or neoplas* or adenocarcinoma* or carcinoma* or malignan* or tumour* or tumor* or blastoma* or metast*)).tw,kf. 3687

13 (pleural adj3 (cancer* or neoplas* or adenocarcinoma* or carcinoma* or malignan* or tumour* or tumor* or blastoma* or metast*)).tw,kf. 19352

14 (Malignant Mesothelioma or Multiple Pulmonary Nodules or Pancoast Syndrome or pancoast tumor* or pancoast tumour*).tw,kf. 8918

15 exp ischemic heart disease/ 760667

16 (Myocardial Ischemia* or Myocardial Ischaemia or ischemic heart disease* or ischaemic heart disease* or Acute Coronary Syndrome* or angina* or angor pectori* or myocardial preinfarction syndrome* or stenocardia* or coronary artery insufficiency or coronary artery occlusive disease* or coronary heart disease* or coronary insufficiency or coronary occlusive disease* or ischaemic cardiac disease* or ischaemic cardial disease* or ischaemic cardiopathy or ischemic cardiac disease* or ischemic cardial disease* or ischemic cardiopathy or Coronary Disease* or Coronary Aneurysm* or Coronary Artery Disease* or Coronary Occlusion or Coronary Stenosis or Coronary Restenosis or Coronary Thrombosis or Coronary Vasospasm or Myocardial Infarct* or stemi or cardiovascular stroke* or heart attack*).tw,kf. 681539

17 exp non insulin dependent diabetes mellitus/ 293486

18 (type* adj3 two adj3 diabet*).tw,kf. 1956

19 (type* adj3 "2" adj3 diabet*).tw,kf. 248163

20 (type* adj3 "II" adj3 diabet*).tw,kf. 19994

21 (adult* adj3 onset adj3 diabet*).tw,kf. 1396

22 (Matur* adj3 onset adj3 diabet*).tw,kf. 3569

23 (slow adj3 onset adj3 diabet*).tw,kf. 35

24 (Ketosis resistant adj3 diabet*).tw,kf. 41

25 (stable adj3 diabet*).tw,kf. 1382

26 (Non insulin adj3 dependent adj3 diabet*).tw,kf. 12632

27 (Noninsulin adj3 dependent adj3 diabet*).tw,kf. 1726

28 NIDDM.tw,kf. 8301

29 MODY.tw,kf. 2620

30 "diabet*".m_titl. 538443

31 exp chronic obstructive lung disease/ 154878

32 (chronic obstructive lung disease or chronic airflow obstruction or chronic airway obstruction or chronic obstructive bronchopulmonary disease or chronic obstructive lung disorder or chronic obstructive pulmonary disease or chronic obstructive pulmonary disorder or chronic obstructive respiratory disease or chronic pulmonary obstructive disease or chronic pulmonary obstructive disorder or copd or lung chronic obstructive disease or obstructive chronic lung disease or obstructive chronic pulmonary disease or chronic bronchitis or emphysema).tw,kf. 175927

33 exp dementia/ 408265

34 (Dementia* or demented or amentia* or alzheimer* or Primary Progressive aphasia or mesulam syndrome or Creutzfeldt Jakob Syndrome or creutzfeldt jakob disease or CADASIL or Frontotemporal Lobar Degeneration or pick* disease or lobar atroph* or Huntington* Disease or huntington* chorea or Kluver Bucy Syndrome or Lewy Body Disease* or Lewy Body Disorder*).tw,kf. 390409

35 or/7-34 2809162

36 6 and 35 28137

37 limit 36 to (chapter or conference abstract or conference paper or "conference review" or editorial or letter) 6561

38 36 not 37 21576

39 38 not ((exp animal/ or animal experiment/ or nonhuman/) not (exp human/ or human experiment/)) 19851

40 limit 39 to embase 16527

41 limit 40 to yr="2000 -Current" 16288

**Ovid EBM Reviews - Cochrane Central Register of Controlled Trials <January 2022>**

**Ovid EBM Reviews - Cochrane Database of Systematic Reviews <2005 to March 2, 2022>**

1 artificial intelligence/ or machine learning/ or deep learning/ or supervised machine learning/ or support vector machine/ or unsupervised machine learning/ 400

2 computer heuristics/ or natural language processing/ or neural networks, computer/ 138

3 (artificial intelligence or Machine learning or neural network* or deep learning or supervised learning or unsupervised learning or deep architecture* or computational intelligence or computer reasoning or machine intelligence or support vector machine* or support vector network* or natural language processing or data driven algorithm* or perceptron or random forest* or ensemble learning or reinforcement learning).tw. 4740

4 1 or 2 or 3 4892

5 exp Respiratory Tract Neoplasms/ 8880

6 (lung* adj3 (cancer* or neoplas* or adenocarcinoma* or carcinoma* or malignan* or tumour* or tumor* or blastoma* or metast*)).tw. 23875

7 (pulmonary adj3 (cancer* or neoplas* or adenocarcinoma* or carcinoma* or malignan* or tumour* or tumor* or blastoma* or metast*)).tw. 1123

8 (bronch* adj3 (cancer* or neoplas* or adenocarcinoma* or carcinoma* or malignan* or tumour* or tumor* or blastoma* or metast*)).tw. 946

9 (alveolar adj3 (cancer* or neoplas* or adenocarcinoma* or carcinoma* or malignan* or tumour* or tumor* or blastoma* or metast*)).tw. 33

10 (trachea* adj3 (cancer* or neoplas* or adenocarcinoma* or carcinoma* or malignan* or tumour* or tumor* or blastoma* or metast*)).tw. 47

11 (pleural adj3 (cancer* or neoplas* or adenocarcinoma* or carcinoma* or malignan* or tumour* or tumor* or blastoma* or metast*)).tw. 1286

12 (Malignant Mesothelioma or Multiple Pulmonary Nodules or Pancoast Syndrome or pancoast tumor* or pancoast tumour*).tw. 177

13 exp Myocardial Ischemia/ 29090

14 (Myocardial Ischemia* or Myocardial Ischaemia or ischemic heart disease* or ischaemic heart disease* or Acute Coronary Syndrome* or angina* or angor pectori* or myocardial preinfarction syndrome* or stenocardia* or coronary artery insufficiency or coronary artery occlusive disease* or coronary heart disease* or coronary insufficiency or coronary occlusive disease* or ischaemic cardiac disease* or ischaemic cardial disease* or ischaemic cardiopathy or ischemic cardiac disease* or ischemic cardial disease* or ischemic cardiopathy or Coronary Disease* or Coronary Aneurysm* or Coronary Artery Disease* or Coronary Occlusion or Coronary Stenosis or Coronary Restenosis or Coronary Thrombosis or Coronary Vasospasm or Myocardial Infarct* or stemi or cardiovascular stroke* or heart attack*).tw. 64219

15 Diabetes Mellitus, Type 2/ 19442

16 (type* adj3 two adj3 diabet*).tw. 456

17 (type* adj3 "2" adj3 diabet*).tw. 41555

18 (type* adj3 "II" adj3 diabet*).tw. 2995

19 (adult* adj3 onset adj3 diabet*).tw. 111

20 (Matur* adj3 onset adj3 diabet*).tw. 98

21 (slow adj3 onset adj3 diabet*).tw. 2

22 (Ketosis resistant adj3 diabet*).tw. 1

23 (stable adj3 diabet*).tw. 635

24 (Non insulin adj3 dependent adj3 diabet*).tw. 2130

25 (Noninsulin adj3 dependent adj3 diabet*).tw. 152

26 NIDDM.tw. 1110

27 MODY.tw. 57

28 "diabet*".m_titl. 62872

29 exp Pulmonary Disease, Chronic Obstructive/ 6146

30 (chronic obstructive lung disease or chronic airflow obstruction or chronic airway obstruction or chronic obstructive bronchopulmonary disease or chronic obstructive lung disorder or chronic obstructive pulmonary disease or chronic obstructive pulmonary disorder or chronic obstructive respiratory disease or chronic pulmonary obstructive disease or chronic pulmonary obstructive disorder or copd or lung chronic obstructive disease or obstructive chronic lung disease or obstructive chronic pulmonary disease or chronic bronchitis or emphysema).tw. 24277

31 exp Dementia/ 6445

32 (Dementia* or demented or amentia* or alzheimer* or Primary Progressive aphasia or mesulam syndrome or Creutzfeldt Jakob Syndrome or creutzfeldt jakob disease or CADASIL or Frontotemporal Lobar Degeneration or pick* disease or lobar atroph* or Huntington* Disease or huntington* chorea or Kluver Bucy Syndrome or Lewy Body Disease* or Lewy Body Disorder*).tw. 23352

33 or/5-32 212821

34 4 and 33 629

35 remove duplicates from 34 616

36 limit 35 to yr="2000 -Current" 578

**Search History**

**Interface - EBSCOhost Research Databases**

**Search Screen - Advanced Search**

**Database - CINAHL Complete**

| \| **#** \| **Query** \| **Limiters/Expanders** \| **Results** \| \| --- \| --- \| --- \| --- \| \| S29 \| S28 NOT ( (((MH "Animals+") OR (MH "Animal Studies") OR (TI "animal model*")) NOT (MH "human")) ) \| Limiters - Published Date: 20000101-20221231 Expanders - Apply equivalent subjects Search modes - Boolean/Phrase \| 2,480 \| \| S28 \| S26 NOT S27 \| Expanders - Apply equivalent subjects Search modes - Boolean/Phrase \| 2,522 \| \| S27 \| S26 \| Limiters - Publication Type: Book, Book Chapter, Book Review, Commentary, Doctoral Dissertation, Editorial, Letter, Masters Thesis, Proceedings, Response Expanders - Apply equivalent subjects Search modes - Boolean/Phrase \| 162 \| \| S26 \| S4 AND S25 \| Expanders - Apply equivalent subjects Search modes - Boolean/Phrase \| 2,684 \| \| S25 \| S5 OR S6 OR S7 OR S8 OR S9 OR S10 OR S11 OR S12 OR S13 OR S14 OR S15 OR S16 OR S17 OR S18 OR S19 OR S20 OR S21 OR S22 OR S23 OR S24 \| Expanders - Apply equivalent subjects Search modes - Boolean/Phrase \| 593,287 \| \| S24 \| (Dementia* or demented or amentia* or alzheimer* or Primary Progressive aphasia or mesulam syndrome or Creutzfeldt Jakob Syndrome or creutzfeldt jakob disease or CADASIL or Frontotemporal Lobar Degeneration or pick* disease or lobar atroph* or Huntington* Disease or huntington* chorea or Kluver Bucy Syndrome or Lewy Body Disease* or Lewy Body Disorder*) \| Expanders - Apply equivalent subjects Search modes - Boolean/Phrase \| 108,849 \| \| S23 \| (MH "Dementia+") \| Expanders - Apply equivalent subjects Search modes - Boolean/Phrase \| 80,378 \| \| S22 \| (chronic obstructive lung disease or chronic airflow obstruction or chronic airway obstruction or chronic obstructive bronchopulmonary disease or chronic obstructive lung disorder or chronic obstructive pulmonary disease or chronic obstructive pulmonary disorder or chronic obstructive respiratory disease or chronic pulmonary obstructive disease or chronic pulmonary obstructive disorder or copd or lung chronic obstructive disease or obstructive chronic lung disease or obstructive chronic pulmonary disease or chronic bronchitis or emphysema) \| Expanders - Apply equivalent subjects Search modes - Boolean/Phrase \| 34,990 \| \| S21 \| (MH "Pulmonary Disease, Chronic Obstructive+") \| Expanders - Apply equivalent subjects Search modes - Boolean/Phrase \| 21,576 \| \| S20 \| NIDDM OR MODY OR TI diabet* \| Expanders - Apply equivalent subjects Search modes - Boolean/Phrase \| 155,249 \| \| S19 \| (stable N3 diabet*) OR (Non insulin N3 dependent N3 diabet*) OR (Noninsulin N3 dependent N3 diabet*) \| Expanders - Apply equivalent subjects Search modes - Boolean/Phrase \| 1,795 \| \| S18 \| (Matur* N3 onset N3 diabet*) OR (slow N3 onset N3 diabet*) OR (Ketosis resistant N3 diabet*) \| Expanders - Apply equivalent subjects Search modes - Boolean/Phrase \| 520 \| \| S17 \| (type* N3 "2" N3 diabet*) OR (type* N3 "II" N3 diabet*) OR (adult* N3 onset N3 diabet*) \| Expanders - Apply equivalent subjects Search modes - Boolean/Phrase \| 86,450 \| \| S16 \| (type* N3 two N3 diabet*) \| Expanders - Apply equivalent subjects Search modes - Boolean/Phrase \| 85,696 \| \| S15 \| (MH "Diabetes Mellitus, Type 2") \| Expanders - Apply equivalent subjects Search modes - Boolean/Phrase \| 67,538 \| \| S14 \| (Myocardial Ischemia* or Myocardial Ischaemia or ischemic heart disease* or ischaemic heart disease* or Acute Coronary Syndrome* or angina* or angor pectori* or myocardial preinfarction syndrome* or stenocardia* or coronary artery insufficiency or coronary artery occlusive disease* or coronary heart disease* or coronary insufficiency or coronary occlusive disease* or ischaemic cardiac disease* or ischaemic cardial disease* or ischaemic cardiopathy or ischemic cardiac disease* or ischemic cardial disease* or ischemic cardiopathy or Coronary Disease* or Coronary Aneurysm* or Coronary Artery Disease* or Coronary Occlusion or Coronary Stenosis or Coronary Restenosis or Coronary Thrombosis or Coronary Vasospasm or Myocardial Infarct* or stemi or cardiovascular stroke* or heart attack*) \| Expanders - Apply equivalent subjects Search modes - Boolean/Phrase \| 210,619 \| \| S13 \| (MH "Myocardial Ischemia+") \| Expanders - Apply equivalent subjects Search modes - Boolean/Phrase \| 110,680 \| \| S12 \| (Malignant Mesothelioma or Multiple Pulmonary Nodules or Pancoast Syndrome or pancoast tumor* or pancoast tumour*) \| Expanders - Apply equivalent subjects Search modes - Boolean/Phrase \| 2,432 \| \| S11 \| (pleural N3 (cancer* or neoplas* or adenocarcinoma* or carcinoma* or malignan* or tumour* or tumor* or blastoma* or metast*)) \| Expanders - Apply equivalent subjects Search modes - Boolean/Phrase \| 3,420 \| \| S10 \| (trachea* N3 (cancer* or neoplas* or adenocarcinoma* or carcinoma* or malignan* or tumour* or tumor* or blastoma* or metast*)) \| Expanders - Apply equivalent subjects Search modes - Boolean/Phrase \| 323 \| \| S9 \| (alveolar N3 (cancer* or neoplas* or adenocarcinoma* or carcinoma* or malignan* or tumour* or tumor* or blastoma* or metast*)) \| Expanders - Apply equivalent subjects Search modes - Boolean/Phrase \| 234 \| \| S8 \| (bronch* N3 (cancer* or neoplas* or adenocarcinoma* or carcinoma* or malignan* or tumour* or tumor* or blastoma* or metast*)) \| Expanders - Apply equivalent subjects Search modes - Boolean/Phrase \| 1,886 \| \| S7 \| (pulmonary N3 (cancer* or neoplas* or adenocarcinoma* or carcinoma* or malignan* or tumour* or tumor* or blastoma* or metast*)) \| Expanders - Apply equivalent subjects Search modes - Boolean/Phrase \| 4,683 \| \| S6 \| (lung* N3 (cancer* or neoplas* or adenocarcinoma* or carcinoma* or malignan* or tumour* or tumor* or blastoma* or metast*)) \| Expanders - Apply equivalent subjects Search modes - Boolean/Phrase \| 70,584 \| \| S5 \| (MH "Respiratory Tract Neoplasms+") \| Expanders - Apply equivalent subjects Search modes - Boolean/Phrase \| 57,268 \| \| S4 \| S1 OR S2 OR S3 \| Expanders - Apply equivalent subjects Search modes - Boolean/Phrase \| 29,395 \| \| S3 \| TI AI \| Expanders - Apply equivalent subjects Search modes - Boolean/Phrase \| 1,646 \| \| S2 \| (artificial intelligence or Machine learning or neural network* or deep learning or supervised learning or unsupervised learning or deep architecture* or computational intelligence or computer reasoning or machine intelligence or support vector machine* or support vector network* or natural language processing or data driven algorithm* or perceptron or random forest* or ensemble learning or reinforcement learning) \| Expanders - Apply equivalent subjects Search modes - Boolean/Phrase \| 28,219 \| \| S1 \| (MH "Artificial Intelligence") OR (MH "Expert Systems") OR (MH "Knowbots") OR (MH "Machine Learning+") OR (MH "Natural Language Processing") OR (MH "Neural Networks (Computer)") \| Expanders - Apply equivalent subjects Search modes - Boolean/Phrase \| 13,842 \| |
| --- | --- | --- | --- | --- | --- | --- | --- | --- | --- | --- | --- | --- | --- | --- | --- | --- | --- | --- | --- | --- | --- | --- | --- | --- | --- | --- | --- | --- | --- | --- | --- | --- | --- | --- | --- | --- | --- | --- | --- | --- | --- | --- | --- | --- | --- | --- | --- | --- | --- | --- | --- | --- | --- | --- | --- | --- | --- | --- | --- | --- | --- | --- | --- | --- | --- | --- | --- | --- | --- | --- | --- | --- | --- | --- | --- | --- | --- | --- | --- | --- | --- | --- | --- | --- | --- | --- | --- | --- | --- | --- | --- | --- | --- | --- | --- | --- | --- | --- | --- | --- | --- | --- | --- | --- | --- | --- | --- | --- | --- | --- | --- | --- | --- | --- | --- | --- | --- | --- | --- | --- |

**Scopus**

**5,878 document results**

( ( ( TITLE-ABS-KEY ( ( "artificial intelligence" OR "Machine learning" OR "neural network* " OR "deep learning " OR "supervised learning " OR "unsupervised learning " OR "deep architecture* " OR "computational intelligence " OR "computer reasoning " OR "machine intelligence " OR "support vect machine*" OR "support vector network* " OR "natural language processing " OR "data driven algorithm* " OR perceptron OR "random forest* " OR "ensemble learning " OR "reinforcement learning" ) ) OR TITLE ( ai ) ) ) AND ( ( ( TITLE-ABS-KEY ( ( lung* W/3 ( cancer* OR neoplas* OR adenocarcinoma* OR carcinoma* OR malignan* OR tumour* OR tumor* OR blastoma* OR metast* ) ) ) ) OR ( TITLE-ABS-KEY ( ( lung* W/3 ( cancer* OR neoplas* OR adenocarcinoma* OR carcinoma* OR malignan* OR tumour* OR tumor* OR blastoma* OR metast* ) ) ) ) OR ( TITLE-ABS-KEY ( ( pulmonary* W/3 ( cancer* OR neoplas* OR adenocarcinoma* OR carcinoma* OR malignan* OR tumour* OR tumor* OR blastoma* OR metast* ) ) ) ) OR ( TITLE-ABS-KEY ( ( bronch* W/3 ( cancer* OR neoplas* OR adenocarcinoma* OR carcinoma* OR malignan* OR tumour* OR tumor* OR blastoma* OR metast* ) ) ) ) OR ( TITLE-ABS-KEY ( ( alveolar W/3 ( cancer* OR neoplas* OR adenocarcinoma* OR carcinoma* OR malignan* OR tumour* OR tumor* OR blastoma* OR metast* ) ) ) ) OR ( TITLE-ABS-KEY ( ( trachea* W/3 ( cancer* OR neoplas* OR adenocarcinoma* OR carcinoma* OR malignan* OR tumour* OR tumor* OR blastoma* OR metast* ) ) ) ) OR ( TITLE-ABS-KEY ( ( pleural W/3 ( cancer* OR neoplas* OR adenocarcinoma* OR carcinoma* OR malignan* OR tumour* OR tumor* OR blastoma* OR metast* ) ) ) ) OR ( TITLE-ABS-KEY ( ( "Malignant Mesothelioma" OR "Multiple Pulmonary Nodules" OR "Pancoast Syndrome" OR "pancoast tumor*" OR "pancoast tumour*" ) ) ) OR ( TITLE-ABS-KEY ( ( "Myocardial Ischemia*" OR "Myocardial Ischaemia" OR "ischemic heart disease*" OR "ischaemic heart disease*" OR "Acute Coronary Syndrome*" OR angina* OR "angor pectori*" OR "myocardial preinfarction syndrome*" OR stenocardia* OR "coronary artery insufficiency" OR "coronary artery occlusive disease*" OR "coronary heart disease*" OR "coronary insufficiency" OR "coronary occlusive disease*" OR "ischaemic cardiac disease*" OR "ischaemic cardial disease*" OR "ischaemic cardiopathy" OR "ischemic cardiac disease*" OR "ischemic cardial disease*" OR "ischemic cardiopathy" OR "Coronary Disease*" OR "Coronary Aneurysm*" OR "Coronary Artery Disease*" OR "Coronary Occlusion" OR "Coronary Stenosis" OR "Coronary Restenosis" OR "Coronary Thrombosis" OR "Coronary Vasospasm" OR "Myocardial Infarct*" OR stemi OR "cardiovascular stroke*" OR "heart attack*" ) ) ) ) OR ( ( TITLE-ABS-KEY ( ( "chronic obstructive lung disease*" OR "chronic airflow obstruction" OR "chronic airway obstruction" OR "chronic obstructive bronchopulmonary disease*" OR "chronic obstructive lung disorder*" OR "chronic obstructive pulmonary disease*" OR "chronic obstructive pulmonary disorder*" OR "chronic obstructive respiratory disease*" OR "chronic pulmonary obstructive disease*" OR "chronic pulmonary obstructive disorder*" OR copd OR "lung chronic obstructive disease*" OR "obstructive chronic lung disease*" OR "obstructive chronic pulmonary disease*" OR "chronic bronchitis" OR emphysema ) ) ) OR ( TITLE-ABS-KEY ( ( dementia* OR demented OR amentia* OR alzheimer* OR "Primary Progressive aphasia" OR "mesulam syndrome" OR "Creutzfeldt Jakob Syndrome" OR "creutzfeldt jakob disease" OR cadasil OR "Frontotemporal Lobar Degeneration" OR "pick* disease" OR "lobar atroph*" OR "Huntington* Disease" OR "huntington* chorea" OR "Kluver Bucy Syndrome" OR "Lewy Body Disease*" OR "Lewy Body Disorder*" ) ) ) OR ( TITLE-ABS-KEY ( ( type* W/3 two W/3 diabet* ) ) ) OR ( TITLE-ABS-KEY ( ( type* W/3 "2" W/3 diabet* ) ) ) OR ( TITLE-ABS-KEY ( ( type* W/3 "II" W/3 diabet* ) ) ) OR ( TITLE-ABS-KEY ( ( adult* W/3 onset W/3 diabet* ) ) ) OR ( TITLE-ABS-KEY ( ( matur* W/3 onset W/3 diabet* ) ) ) ) OR ( ( TITLE-ABS-KEY ( ( slow W/3 onset W/3 diabet* ) ) ) OR ( TITLE-ABS-KEY ( ( "Ketosis resistant" W/3 diabet* ) ) ) OR ( TITLE-ABS-KEY ( ( stable W/3 diabet* ) ) ) OR ( TITLE-ABS-KEY ( ( "Non insulin" W/3 dependent W/3 diabet* ) ) ) ) OR ( ( TITLE-ABS-KEY ( ( noninsulin W/3 dependent W/3 diabet* ) ) ) OR ( TITLE-ABS-KEY ( niddm ) ) ) OR ( ( TITLE-ABS-KEY ( mody ) ) OR ( TITLE ( diabet* ) ) ) ) ) AND NOT ( TITLE ( animal* OR nonhuman* OR veterinar* OR avian* OR baboon* OR bird* OR bovine OR canine OR cat OR cats OR cattle* OR chick* OR chimp* OR cow OR cows OR dog OR dogs OR duck OR feline OR fish* OR geese OR goose OR macaque* OR marmoset* OR mice OR mouse OR murine OR ovine OR pig OR pigs OR piglet* OR porcine OR primate* OR rabbit OR rat OR rats OR rodent* OR sheep OR swine OR trout* OR zebrafish* ) AND NOT ( human* OR patient* OR women OR woman OR men OR man ) ) AND NOT INDEX ( medline ) AND ( EXCLUDE ( DOCTYPE , "cp" ) OR EXCLUDE ( DOCTYPE , "cr" ) OR EXCLUDE ( DOCTYPE , "ed" ) OR EXCLUDE ( DOCTYPE , "ch" ) OR EXCLUDE ( DOCTYPE , "le" ) OR EXCLUDE ( DOCTYPE , "bk" ) ) AND ( LIMIT-TO ( PUBYEAR , 2022 ) OR LIMIT-TO ( PUBYEAR , 2021 ) OR LIMIT-TO ( PUBYEAR , 2020 ) OR LIMIT-TO ( PUBYEAR , 2019 ) OR LIMIT-TO ( PUBYEAR , 2018 ) OR LIMIT-TO ( PUBYEAR , 2017 ) OR LIMIT-TO ( PUBYEAR , 2016 ) OR LIMIT-TO ( PUBYEAR , 2015 ) OR LIMIT-TO ( PUBYEAR , 2014 ) OR LIMIT-TO ( PUBYEAR , 2013 ) OR LIMIT-TO ( PUBYEAR , 2012 ) OR LIMIT-TO ( PUBYEAR , 2011 ) OR LIMIT-TO ( PUBYEAR , 2010 ) OR LIMIT-TO ( PUBYEAR , 2009 ) OR LIMIT-TO ( PUBYEAR , 2008 ) OR LIMIT-TO ( PUBYEAR , 2007 ) OR LIMIT-TO ( PUBYEAR , 2006 ) OR LIMIT-TO ( PUBYEAR , 2005 ) OR LIMIT-TO ( PUBYEAR , 2004 ) OR LIMIT-TO ( PUBYEAR , 2003 ) OR LIMIT-TO ( PUBYEAR , 2002 ) OR LIMIT-TO ( PUBYEAR , 2001 ) OR LIMIT-TO ( PUBYEAR , 2000 ) )

**Web of Science Selected Databases, searched simultaneously:**

**Science Citation Index Expanded**

**Social Sciences Citation Index**

**Emerging Sources Citation Index**

Editions = ESCI , SCI-EXPANDED , SSCI

| Query | Search History | Results |
| --- | --- | --- |
| 1 | (TI=("artificial intelligence" OR "Machine learning" OR "neural network* " OR "deep learning " OR "supervised learning " OR "unsupervised learning " OR "deep architecture* " OR "computational intelligence " OR "computer reasoning " OR "machine intelligence " OR "support vector machine*" OR "support vector network* " OR "natural language processing " OR "data driven algorithm* " OR perceptron OR "random forest* " OR "ensemble learning " OR "reinforcement learning") OR AB=("artificial intelligence" OR "Machine learning" OR "neural network* " OR "deep learning " OR "supervised learning " OR "unsupervised learning " OR "deep architecture* " OR "computational intelligence " OR "computer reasoning " OR "machine intelligence " OR "support vect machine*" OR "support vector network* " OR "natural language processing " OR "data driven algorithm* " OR perceptron OR "random forest* " OR "ensemble learning " OR "reinforcement learning") OR TI=(AI) ) | 464,227 |
| 2 | (TI=("artificial intelligence" OR "Machine learning" OR "neural network* " OR "deep learning " OR "supervised learning " OR "unsupervised learning " OR "deep architecture* " OR "computational intelligence " OR "computer reasoning " OR "machine intelligence " OR "support vector machine*" OR "support vector network* " OR "natural language processing " OR "data driven algorithm* " OR perceptron OR "random forest* " OR "ensemble learning " OR "reinforcement learning") OR AB=("artificial intelligence" OR "Machine learning" OR "neural network* " OR "deep learning " OR "supervised learning " OR "unsupervised learning " OR "deep architecture* " OR "computational intelligence " OR "computer reasoning " OR "machine intelligence " OR "support vector machine*" OR "support vector network* " OR "natural language processing " OR "data driven algorithm* " OR perceptron OR "random forest* " OR "ensemble learning " OR "reinforcement learning") OR TI=(AI) ) NOT TI=( animal* OR nonhuman* OR veterinar* OR avian* OR baboon* OR bird* OR bovine OR canine OR cat OR cats OR cattle* OR chick* OR chimp* OR cow OR cows OR dog OR dogs OR duck OR feline OR fish* OR geese OR goose OR macaque* OR marmoset* OR mice OR mouse OR murine OR ovine OR pig OR pigs OR piglet* OR porcine OR primate* OR rabbit OR rat OR rats OR rodent* OR sheep OR swine OR trout* OR zebrafish* ) | 458,786 |
| 3 | ((TI=("artificial intelligence" OR "Machine learning" OR "neural network* " OR "deep learning " OR "supervised learning " OR "unsupervised learning " OR "deep architecture* " OR "computational intelligence " OR "computer reasoning " OR "machine intelligence " OR "support vector machine*" OR "support vector network* " OR "natural language processing " OR "data driven algorithm* " OR perceptron OR "random forest* " OR "ensemble learning " OR "reinforcement learning") OR AB=("artificial intelligence" OR "Machine learning" OR "neural network* " OR "deep learning " OR "supervised learning " OR "unsupervised learning " OR "deep architecture* " OR "computational intelligence " OR "computer reasoning " OR "machine intelligence " OR "support vector machine*" OR "support vector network* " OR "natural language processing " OR "data driven algorithm* " OR perceptron OR "random forest* " OR "ensemble learning " OR "reinforcement learning") OR TI=(AI) ) NOT TI=( animal* OR nonhuman* OR veterinar* OR avian* OR baboon* OR bird* OR bovine OR canine OR cat OR cats OR cattle* OR chick* OR chimp* OR cow OR cows OR dog OR dogs OR duck OR feline OR fish* OR geese OR goose OR macaque* OR marmoset* OR mice OR mouse OR murine OR ovine OR pig OR pigs OR piglet* OR porcine OR primate* OR rabbit OR rat OR rats OR rodent* OR sheep OR swine OR trout* OR zebrafish* )) AND TS=(("Myocardial Ischemia*" or "Myocardial Ischaemia" or "ischemic heart disease*" or "ischaemic heart disease*" or "Acute Coronary Syndrome*" or angina* or "angor pectori*" or "myocardial preinfarction syndrome*" or stenocardia* or "coronary artery insufficiency" or "coronary artery occlusive disease*" or "coronary heart disease*" or "coronary insufficiency" or "coronary occlusive disease*" or "ischaemic cardiac disease*" or "ischaemic cardial disease*" or "ischaemic cardiopathy" or "ischemic cardiac disease*" or "ischemic cardial disease*" or "ischemic cardiopathy" or "Coronary Disease*" or "Coronary Aneurysm*" or "Coronary Artery Disease*" or "Coronary Occlusion" or "Coronary Stenosis" or "Coronary Restenosis" or "Coronary Thrombosis" or "Coronary Vasospasm" or "Myocardial Infarct*" or stemi or "cardiovascular stroke*" or "heart attack*") ) and Articles or Review Articles or Early Access or Corrections or Data Papers (Document Types)  Timespan: 2000-01-01 to 2022-12-31 (Publication Date) | 1,833 |
| 4 | ((TI=("artificial intelligence" OR "Machine learning" OR "neural network* " OR "deep learning " OR "supervised learning " OR "unsupervised learning " OR "deep architecture* " OR "computational intelligence " OR "computer reasoning " OR "machine intelligence " OR "support vector machine*" OR "support vector network* " OR "natural language processing " OR "data driven algorithm* " OR perceptron OR "random forest* " OR "ensemble learning " OR "reinforcement learning") OR AB=("artificial intelligence" OR "Machine learning" OR "neural network* " OR "deep learning " OR "supervised learning " OR "unsupervised learning " OR "deep architecture* " OR "computational intelligence " OR "computer reasoning " OR "machine intelligence " OR "support vector machine*" OR "support vector network* " OR "natural language processing " OR "data driven algorithm* " OR perceptron OR "random forest* " OR "ensemble learning " OR "reinforcement learning") OR TI=(AI) ) NOT TI=( animal* OR nonhuman* OR veterinar* OR avian* OR baboon* OR bird* OR bovine OR canine OR cat OR cats OR cattle* OR chick* OR chimp* OR cow OR cows OR dog OR dogs OR duck OR feline OR fish* OR geese OR goose OR macaque* OR marmoset* OR mice OR mouse OR murine OR ovine OR pig OR pigs OR piglet* OR porcine OR primate* OR rabbit OR rat OR rats OR rodent* OR sheep OR swine OR trout* OR zebrafish* )) AND TS=(("chronic obstructive lung disease*" or "chronic airflow obstruction" or "chronic airway obstruction" or "chronic obstructive bronchopulmonary disease*" or "chronic obstructive lung disorder*" or "chronic obstructive pulmonary disease*" or "chronic obstructive pulmonary disorder*" or "chronic obstructive respiratory disease*" or "chronic pulmonary obstructive disease*" or "chronic pulmonary obstructive disorder*" or copd or "lung chronic obstructive disease*" or "obstructive chronic lung disease*" or "obstructive chronic pulmonary disease*" or "chronic bronchitis" or emphysema) ) and Articles or Review Articles or Early Access or Corrections (Document Types)  Timespan: 2000-01-01 to 2022-12-31 (Publication Date) | 426 |
| 5 | ((TI=("artificial intelligence" OR "Machine learning" OR "neural network* " OR "deep learning " OR "supervised learning " OR "unsupervised learning " OR "deep architecture* " OR "computational intelligence " OR "computer reasoning " OR "machine intelligence " OR "support vector machine*" OR "support vector network* " OR "natural language processing " OR "data driven algorithm* " OR perceptron OR "random forest* " OR "ensemble learning " OR "reinforcement learning") OR AB=("artificial intelligence" OR "Machine learning" OR "neural network* " OR "deep learning " OR "supervised learning " OR "unsupervised learning " OR "deep architecture* " OR "computational intelligence " OR "computer reasoning " OR "machine intelligence " OR "support vector machine*" OR "support vector network* " OR "natural language processing " OR "data driven algorithm* " OR perceptron OR "random forest* " OR "ensemble learning " OR "reinforcement learning") OR TI=(AI) ) NOT TI=( animal* OR nonhuman* OR veterinar* OR avian* OR baboon* OR bird* OR bovine OR canine OR cat OR cats OR cattle* OR chick* OR chimp* OR cow OR cows OR dog OR dogs OR duck OR feline OR fish* OR geese OR goose OR macaque* OR marmoset* OR mice OR mouse OR murine OR ovine OR pig OR pigs OR piglet* OR porcine OR primate* OR rabbit OR rat OR rats OR rodent* OR sheep OR swine OR trout* OR zebrafish* )) AND TS=((Dementia* or demented or amentia* or alzheimer* or "Primary Progressive aphasia" or "mesulam syndrome" or "Creutzfeldt Jakob Syndrome" or "creutzfeldt jakob disease" or CADASIL or "Frontotemporal Lobar Degeneration" or "pick* disease" or "lobar atroph*" or "Huntington* Disease" or "huntington* chorea" or "Kluver Bucy Syndrome" or "Lewy Body Disease*" or "Lewy Body Disorder*") ) and Articles or Review Articles or Early Access or Corrections or Data Papers (Document Types)  Timespan: 2000-01-01 to 2022-12-31 (Publication Date) | 3,460 |
| 6 | ((TI=("artificial intelligence" OR "Machine learning" OR "neural network* " OR "deep learning " OR "supervised learning " OR "unsupervised learning " OR "deep architecture* " OR "computational intelligence " OR "computer reasoning " OR "machine intelligence " OR "support vector machine*" OR "support vector network* " OR "natural language processing " OR "data driven algorithm* " OR perceptron OR "random forest* " OR "ensemble learning " OR "reinforcement learning") OR AB=("artificial intelligence" OR "Machine learning" OR "neural network* " OR "deep learning " OR "supervised learning " OR "unsupervised learning " OR "deep architecture* " OR "computational intelligence " OR "computer reasoning " OR "machine intelligence " OR "support vector machine*" OR "support vector network* " OR "natural language processing " OR "data driven algorithm* " OR perceptron OR "random forest* " OR "ensemble learning " OR "reinforcement learning") OR TI=(AI) ) NOT TI=( animal* OR nonhuman* OR veterinar* OR avian* OR baboon* OR bird* OR bovine OR canine OR cat OR cats OR cattle* OR chick* OR chimp* OR cow OR cows OR dog OR dogs OR duck OR feline OR fish* OR geese OR goose OR macaque* OR marmoset* OR mice OR mouse OR murine OR ovine OR pig OR pigs OR piglet* OR porcine OR primate* OR rabbit OR rat OR rats OR rodent* OR sheep OR swine OR trout* OR zebrafish* )) AND (TI=(diabet* ) OR AB=(Diabet*)) and Articles or Review Articles or Early Access or Corrections or Data Papers (Document Types)  Timespan: 2000-01-01 to 2022-12-31 (Publication Date) | 3,287 |
| 7 | "lung* cancer*" or "lung neoplas*" or "lung adenocarcinoma*" or "lung carcinoma*" or "lung malignan*" or "lung tumour*" or "lung tumour*" or "lung blastoma*" or "lung metast*" or "pulmonary cancer*" or "pulmonary neoplas*" or "pulmonary adenocarcinoma*" or "pulmonary carcinoma*" or "pulmonary malignan*" or "pulmonary tumour*" or "pulmonary tumor "pulmonary *" or "pulmonary blastoma*" or "pulmonary metast*" or Mesothelioma or "bronch* cancer*" or "bronch* neoplas*" or "bronch* adenocarcinoma*" or "bronch* carcinoma*" or "bronch* malignan*" or "bronch* tumour*" or "bronch* tumour*" or "bronch* blastoma*" or "bronch* metast*" or "trachea* cancer*" or "trachea* neoplas*" or "trachea* adenocarcinoma*" or "trachea* carcinoma*" or "trachea* malignan*" or "trachea* tumour*" or "trachea* tumour*" or "trachea* blastoma*" or "trachea* metast*" or "alveolar * cancer*" or "alveolar neoplas*" or "alveolar adenocarcinoma*" or "alveolar carcinoma*" or "alveolar malignan*" or "alveolar tumour*" or "alveolar tumour*" or "alveolar blastoma*" or "alveolar metast*" (Title) or "lung* cancer*" or "lung neoplas*" or "lung adenocarcinoma*" or "lung carcinoma*" or "lung malignan*" or "lung tumour*" or "lung tumour*" or "lung blastoma*" or "lung metast*" or "pulmonary cancer*" or "pulmonary neoplas*" or "pulmonary adenocarcinoma*" or "pulmonary carcinoma*" or "pulmonary malignan*" or "pulmonary tumour*" or "pulmonary tumor "pulmonary *" or "pulmonary blastoma*" or "pulmonary metast*" or Mesothelioma or "bronch* cancer*" or "bronch* neoplas*" or "bronch* adenocarcinoma*" or "bronch* carcinoma*" or "bronch* malignan*" or "bronch* tumour*" or "bronch* tumour*" or "bronch* blastoma*" or "bronch* metast*" or "trachea* cancer*" or "trachea* neoplas*" or "trachea* adenocarcinoma*" or "trachea* carcinoma*" or "trachea* malignan*" or "trachea* tumour*" or "trachea* tumour*" or "trachea* blastoma*" or "trachea* metast*" or "alveolar * cancer*" or "alveolar neoplas*" or "alveolar adenocarcinoma*" or "alveolar carcinoma*" or "alveolar malignan*" or "alveolar tumour*" or "alveolar tumour*" or "alveolar blastoma*" or "alveolar metast*" (Abstract) | 175,428 |
| 8 | #7 AND #2 and Articles or Review Articles or Early Access or Corrections or Retracted Publications (Document Types)  Timespan: 2000-01-01 to 2022-12-31 (Publication Date) | 951 |
|  | TOTAL RESULTS Saved from Queries 3, 4, 5, 6, 8 | 9,957 |

**ACM Digital Library (Association for Computing Machinery)**

[[Abstract: "artificial intelligence"] OR [Abstract: "machine learning"] OR [Abstract: "neural network* "] OR [Abstract: "deep learning "] OR [Abstract: "supervised learning "] OR [Abstract: "unsupervised learning "] OR [Abstract: "deep architecture* "] OR [Abstract: "computational intelligence "] OR [Abstract: "computer reasoning "] OR [Abstract: "machine intelligence "] OR [Abstract: "support vector machine*"] OR [Abstract: "support vector network* "] OR [Abstract: "natural language processing "] OR [Abstract: "data driven algorithm* "] OR [Abstract: perceptron] OR [Abstract: "random forest* "] OR [Abstract: "ensemble learning "] OR [Abstract: "reinforcement learning"]] AND [[Abstract: "lung cancer*"] OR [Abstract: or heart or coronary or alzheimer* or dementia* or] OR [Abstract: "chronic obstructive lung disease*"] OR [Abstract: diabet*] OR [Abstract: or copd]] AND [Publication Date: (01/01/2000 TO 30/04/2022)]

[[Title: "artificial intelligence"] OR [Title: "machine learning"] OR [Title: "neural network* "] OR [Title: "deep learning "] OR [Title: "supervised learning "] OR [Title: "unsupervised learning "] OR [Title: "deep architecture* "] OR [Title: "computational intelligence "] OR [Title: "computer reasoning "] OR [Title: "machine intelligence "] OR [Title: "support vector machine*"] OR [Title: "support vector network* "] OR [Title: "natural language processing "] OR [Title: "data driven algorithm* "] OR [Title: perceptron] OR [Title: "random forest* "] OR [Title: "ensemble learning "] OR [Title: "reinforcement learning"]] AND [[Title: "lung cancer*"] OR [Title: or heart or coronary or alzheimer* or dementia* or] OR [Title: "chronic obstructive lung disease*"] OR [Title: diabet*] OR [Title: or copd]] AND [Publication Date: (01/01/2000 TO 30/04/2022)]

Limit to Journals

66 records

**Inspec (Engineering Village/Elsevier)**

| **#** | **Query** | **Databases** | **Number of results** |
| --- | --- | --- | --- |
| 3 | (( ((({lung cancer*} OR {lung neoplas*} OR alzheimer* OR dementia* OR {chronic obstructive lung disease*} OR {chronic obstructive pulmonary disease*} OR diabet* OR $copd OR {Myocardial Ischemia*} OR {Myocardial Ischaemia} OR {ischemic heart disease*} OR {Acute Coronary Syndrome*} OR angina* OR {Coronary Disease*} OR {Myocardial Infarct*} OR {heart attack*})) WN KY) AND (JA WN DT) AND (2000-2022 WN YR)) AND ( ((({artificial intelligence} OR {machine learning} OR {neural network*} OR {deep learning} OR {supervised learning} OR {unsupervised learning} OR {deep architecture*} OR {computational intelligence} OR {computer reasoning} OR {machine intelligence} OR {support vector machine*} OR {support vector network*} OR {natural language processing} OR {data driven algorithm*} OR $perceptron OR {random forest*} OR {ensemble learning} OR {reinforcement learning})) WN ALL) AND (JA WN DT) AND (2000-2022 WN YR))) | Inspec | 3399 |
| 2 | ((( {artificial intelligence} OR {machine learning} OR {neural network*} OR {deep learning} OR {supervised learning} OR {unsupervised learning} OR {deep architecture*} OR {computational intelligence} OR {computer reasoning} OR {machine intelligence} OR {support vector machine*} OR {support vector network*} OR {natural language processing} OR {data driven algorithm*} OR perceptron OR {random forest*} OR {ensemble learning} OR {reinforcement learning})) WN ALL) AND (JA WN DT) | Inspec | 401049 |
| 1 | ((({lung cancer*} OR {lung neoplas*} OR alzheimer* OR dementia* OR {chronic obstructive lung disease*} OR {chronic obstructive pulmonary disease*} OR diabet* OR copd OR {Myocardial Ischemia*} OR {Myocardial Ischaemia} OR {ischemic heart disease*} OR {Acute Coronary Syndrome*} OR angina* OR {Coronary Disease*} OR {Myocardial Infarct*} OR {heart attack*})) WN KY) AND (JA WN DT) | Inspec | 21040 |
